# Conformational Changes during Pore Formation by the Perforin-Related Protein Pleurotolysin

**DOI:** 10.1371/journal.pbio.1002049

**Published:** 2015-02-05

**Authors:** Natalya Lukoyanova, Stephanie C. Kondos, Irene Farabella, Ruby H. P. Law, Cyril F. Reboul, Tom T. Caradoc-Davies, Bradley A. Spicer, Oded Kleifeld, Daouda A. K. Traore, Susan M. Ekkel, Ilia Voskoboinik, Joseph A. Trapani, Tamas Hatfaludi, Katherine Oliver, Eileen M. Hotze, Rodney K. Tweten, James C. Whisstock, Maya Topf, Helen R. Saibil, Michelle A. Dunstone

**Affiliations:** 1 Department of Crystallography/Biological Sciences, Institute of Structural and Molecular Biology, Birkbeck College, London, United Kingdom; 2 Department of Biochemistry and Molecular Biology, Monash University, Clayton, Melbourne, Australia; 3 The ARC Centre of Excellence in Advanced Molecular Imaging, Monash University, Melbourne, Australia; 4 Australian Synchrotron, Clayton, Melbourne, Australia; 5 Peter MacCallum Cancer Centre, East Melbourne, Australia; 6 Institute of Structural and Molecular Biology, University College London, London, United Kingdom; 7 Department of Microbiology and Immunology, The University of Oklahoma Health Sciences Center, Oklahoma City, Oklahoma, United States of America; 8 Department of Microbiology, Monash University, Melbourne, Australia; University of Zurich, SWITZERLAND

## Abstract

Membrane attack complex/perforin-like (MACPF) proteins comprise the largest superfamily of pore-forming proteins, playing crucial roles in immunity and pathogenesis. Soluble monomers assemble into large transmembrane pores via conformational transitions that remain to be structurally and mechanistically characterised. Here we present an 11 Å resolution cryo-electron microscopy (cryo-EM) structure of the two-part, fungal toxin Pleurotolysin (Ply), together with crystal structures of both components (the lipid binding PlyA protein and the pore-forming MACPF component PlyB). These data reveal a 13-fold pore 80 Å in diameter and 100 Å in height, with each subunit comprised of a PlyB molecule atop a membrane bound dimer of PlyA. The resolution of the EM map, together with biophysical and computational experiments, allowed confident assignment of subdomains in a MACPF pore assembly. The major conformational changes in PlyB are a ∼70° opening of the bent and distorted central β-sheet of the MACPF domain, accompanied by extrusion and refolding of two α-helical regions into transmembrane β-hairpins (TMH1 and TMH2). We determined the structures of three different disulphide bond-trapped prepore intermediates. Analysis of these data by molecular modelling and flexible fitting allows us to generate a potential trajectory of β-sheet unbending. The results suggest that MACPF conformational change is triggered through disruption of the interface between a conserved helix-turn-helix motif and the top of TMH2. Following their release we propose that the transmembrane regions assemble into β-hairpins via top down zippering of backbone hydrogen bonds to form the membrane-inserted β-barrel. The intermediate structures of the MACPF domain during refolding into the β-barrel pore establish a structural paradigm for the transition from soluble monomer to pore, which may be conserved across the whole superfamily. The TMH2 region is critical for the release of both TMH clusters, suggesting why this region is targeted by endogenous inhibitors of MACPF function.

## Author Summary

Animals, plants, fungi, and bacteria all use pore-forming proteins of the membrane attack complex-perforin (MACPF) family as lethal, cell-killing weapons. These proteins are able to insert into the plasma membranes of target cells, creating large pores that short circuit the natural separation between the intracellular and extracellular milieu, with catastrophic results. However, the pore-forming proteins must undergo a substantial transformation from soluble precursors to a large barrel-shaped transmembrane complex as they punch their way into cells. Using a combination of X-ray crystallography and cryo electron microscopy, we have visualized, for the first time, the mechanism of action of one of these pore-forming proteins—pleurotolysin, a MACPF protein from the edible oyster mushroom. This enabled us to propose a model of the pleurotolysin pore by fitting the crystallographic structures of the pore proteins into a three-dimensional map of the pore obtained by cryo electron microscopy. We then designed a set of double mutants that allowed us to chemically trap intermediate states along the trajectory of the pore formation process, and to determine their structures too. By combining these data we proposed a detailed molecular mechanism for pore formation. The pleurotolysin first assembles into rings of 13 subunits, each of which then opens up by about 70° during pore formation. This process is accompanied by refolding and extrusion of two compact regions from each subunit into long hairpins that then zipper together to form an 80-Å wide barrel-shaped channel through the membrane.

## Abstract 3

A combination of structural methods reveals the complex process by which the perforin-like fungal toxin Pleurotolysin rearranges its structure to form a pore that punches a hole in target cell membranes.

## Introduction

Membrane pore-forming proteins have the unique property of being expressed as metastable, water-soluble monomers that convert into a membrane inserted form. These proteins typically assemble into prepore oligomers on the target membrane surface. A dramatic conformational change then permits membrane insertion and formation of transmembrane pores [1–4].

The membrane attack complex/perforin-like family (MACPF) proteins form the largest superfamily of pore-forming proteins identified to date. They include perforin and complement component-9 (C9), mammalian pore-forming proteins that function as weapons of the humoral and cellular immune system, respectively [5]. The superfamily also includes a wide range of molecules implicated in defense or attack [6–8]. For example, invasion by the protozoan parasites *Plasmodium* spp. and egress by *Toxoplasma gondii* requires MACPF proteins, plants utilize the MACPF fold to combat bacterial infection [9], and MACPF-related proteins can be identified in numerous Gram negative and Gram positive bacteria. Finally, a significant group of MACPF proteins play important, but poorly understood, roles in embryonic development and neurobiology [10–12].

Despite the absence of detectable sequence identity, the first crystal structures of MACPF proteins revealed that the pore-forming domain unexpectedly shared homology with the pore-forming bacterial cholesterol dependent cytolysins (CDCs) family [13–15]. This structural similarity extended across the key elements involved in pore formation (originally annotated as three non-contiguous domains 1–3 in CDCs). The central, common feature of the MACPF/CDC fold is a four stranded, highly twisted β-sheet decorated with three small clusters of α-helices. Two of these helical bundles contain the regions destined to insert into the membrane (transmembrane hairpins TMH1 and TMH2). The third α-helical region comprises a short helix-turn-helix (HTH) motif formed *via* a sequence insertion at the bend of the central β-sheet. The HTH motif packs on top of TMH2. These structural similarities, together with commonality of a pore-forming function, suggested that MACPF proteins share a common ancestor with CDCs and assemble into giant pores via a CDC-like mechanism [13,14,16–19].

Previous studies have provided important insight into pore formation by CDCs. Electron microscopy (EM), biochemical, and biophysical studies of CDCs showed that monomers assemble into prepore oligomers on the membrane surface without major conformational changes in the subunits [17,19–22]. However, conversion to the pore form involves dramatic secondary and tertiary conformational changes in which the highly twisted β-sheet opens up and the assembly collapses ∼40 Å towards the membrane surface, allowing unfurling of TMH1 and TMH2 and their insertion into the membrane as amphipathic β-hairpins [19–22].

The CDCs form initial interactions with the membrane through a C-terminal lipid binding immunoglobulin-like (Ig) domain. In the MACPF branch of the superfamily a wide variety of domains are found both N- and C-terminal to the pore-forming MACPF domain. For example, perforin includes a C-terminal lipid and calcium binding C2 domain (a variation of the Ig fold). Similar to the CDC Ig domain, this region mediates initial interaction of perforin with the target membrane. The MACPF domains in the complement membrane attack complex proteins are flanked by arrays of small disulphide constrained domains (e.g., thrombospondin, epidermal growth factor, and complement control protein domains). Rather than interacting directly with membranes, the role of these regions includes mediation of key protein-protein interactions that recruit the MACPF domain to the target cell surface [23–25].

The molecular structures of key intermediates in the assembly of MACPF and CDC pore complexes remain obscure, but are necessary to understand the transition from a monomeric form into oligomeric membrane prepores and then into pores. Here we have analysed this transition, using a variety of structural and biophysical approaches. Structures of MACPF and CDC oligomeric assemblies by EM have been very limited in resolution, owing to their heterogeneity and flexibility. To gain further insight into the structural conversions in pore formation, we chose pleurotolysin (Ply), a MACPF protein consisting of two components, PlyA and PlyB, from *Pleurotus ostreatus* [26,27]. Previous studies have shown that PlyA binds membranes and is required to recruit the pore-forming MACPF protein PlyB to the membrane surface. PlyA and PlyB together form relatively small and regular pores in liposomes [27,28]. As well as determining the structure of the pleurotolysin pore, we used protein-engineering approaches to trap and structurally characterise three distinct prepore intermediates. Together these approaches allowed us to visualise a potential molecular trajectory of a MACPF protein during pore formation.

## Results

### Crystal Structures of the Pleurotolysin Components

**Figure 1:**
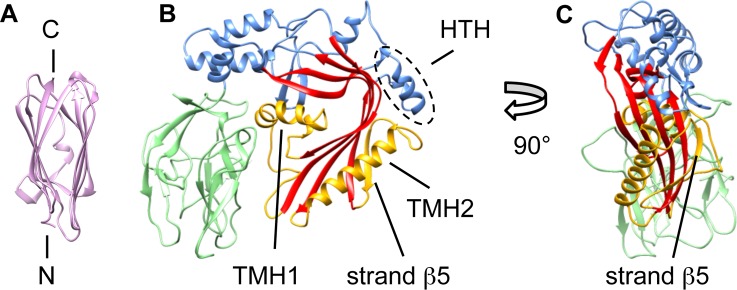
Crystal structures of the two pleurotolysin components: PlyA and PlyB. (A) The structure of PlyA showing a β-sandwich fold similar to that seen in actinoporins [29]. (B) The structure of PlyB, with the bent, central β-sheet characteristic of the MACPF/CDC superfamily (red). The transmembrane hairpin regions are labelled as TMH1 and TMH2 (yellow) and the helix-turn-helix motif is labelled HTH (outlined by the dashed oval). The trefoil of C-terminal β-rich domains is shown in green. The upper part of the central sheet is flanked mainly by helical regions (blue). The conserved pore-forming core consists of the bent β-sheet and the TMH domains. (C) PlyB seen edge-on, clearly showing strand β5.

The 1.85 Å X-ray crystal structure of PlyA (Fig. 1A; S1 Table) revealed a β-sandwich fold, unexpectedly related to the actinoporin-like family of pore-forming toxins [29]. Previous studies suggest that actinoporin-like proteins interact with membranes *via* one end of the β-sandwich, with the N-terminal sequence responsible for forming the pore [29]. However, PlyA lacks the proposed actinoporin N-terminal transmembrane region consistent with the observation that PlyA binds membranes, but is unable to form pores on its own [27].

The 2.2 Å structure of PlyB (Fig. 1B and 1C; S2 Table) reveals an N-terminal MACPF domain (blue/red/yellow) followed by three small β-rich domains clustered in a globular trefoil-like arrangement (green). The MACPF domain of PlyB contains a central, four-stranded bent and twisted β-sheet characteristic of the MACPF/CDC superfamily (red). The TMH1 cluster of helices (yellow) is located on the inside of PlyB, next to the concave face of the central β-sheet. TMH2 (yellow) comprises a single large α-helix and an additional β-strand (termed “strand β5”), located on the edge of the central β-sheet. Together, the central β-sheet and the TMH regions constitute the topologically conserved MACPF/CDC pore-forming fold.

### Cryo-EM Structure of the Pleurotolysin Pore

**Figure 2:**
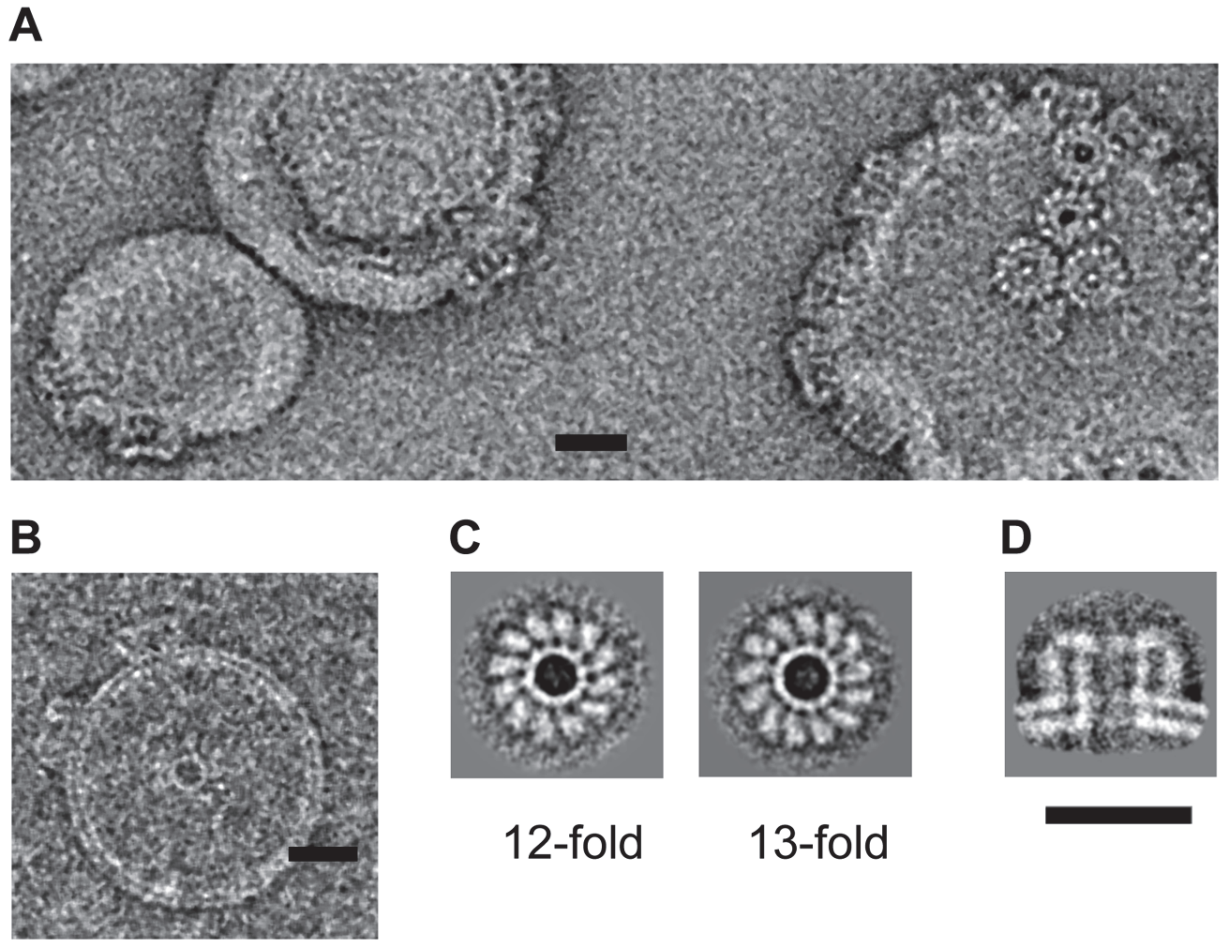
Electron microscopy of pleurotolysin pores. Representative views of negatively stained (A) and vitrified (B) Ply pores on liposomes. (C) Averaged views of 12-fold and 13-fold symmetric pores on lipid monolayers (negative stain). (D) Averaged side view of Ply pores on liposomes (cryo-EM). Scale bar, 20 nm.

**Figure 3:**
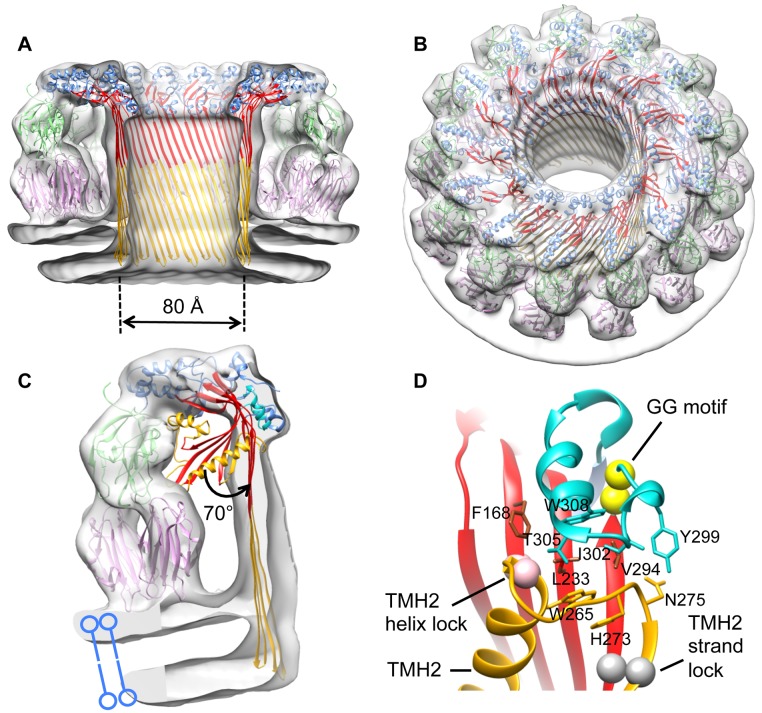
Structure of the pleurotolysin pore. (A) Cut away side and (B) tilted surface views of the cryo-EM reconstruction of a pleurotolysin pore with the fitted atomic structures. (C) Segment of the pore map corresponding to a single subunit with pore model fitted into the density. The PlyB crystal structure is superposed to show a 70° opening of the MACPF β-sheet (red) and movement of the HTH motif (cyan). TMH regions (yellow) are refolded into transmembrane β-hairpins. The PlyB C-terminal trefoil (green) sits on top of the PlyA dimer (pink). (D) Interface between TMH2, the HTH region, and the underlying sheet in the PlyB crystal structure. The position of the TMH2 helix lock (pink spheres) and TMH2 strand lock (grey spheres) are shown. The highly conserved “GG” motif (296–297) in the HTH region is represented as yellow spheres.

EM images of liposomes with added PlyAB showed distinctive, ring shaped pore structures (Fig. 2A and 2B). Analysis of negative stain EM images of oligomeric rings of Ply on membranes showed that the majority of the oligomers had 13-fold symmetry (75%), but 12- (15%), 11- (5%), and 14-fold (5%) rings were also present (Fig. 2C). For 3-D reconstruction, we extracted 14,700 individual cryo-EM images of pore side views in liposomes (Fig. 2D). The images were analysed by the single particle approach, following the method developed for the CDC pneumolysin [17]. This allowed us to sort the pore views by symmetry, enabling determination of an 11 Å resolution cryo-EM map of a liposome-embedded 13-fold pleurotolysin pore from 8,770 views (Fig. 3A and 3B). We used the crystal structures of PlyA and PlyB together with biophysical data (S1 Fig.) to interpret the map. A single PlyB moiety was fitted into the upper part of the pore structure (Fig. 3C). The C-terminal trefoil (green) and the α-helices at the top of the MACPF domain (blue) unambiguously fit the EM density with only minor structural rearrangement. The core of the MACPF domain undergoes a massive opening but does not collapse as in CDCs (Fig. 3C).

The structure was modeled by flexible fitting in a multistep procedure [30]. In the pore map, the position of PlyB is clearly recognizable in the upper part of each subunit, while the V-shaped density at the base of each asymmetric unit accommodates two PlyA molecules. The positions of PlyB subdomains were refined without TMH1 and TMH2, because these transmembrane regions are expected to refold to form the β-barrel of the pore. The best fits were further refined with Flex-EM [30] via simulated annealing rigid-body dynamics.

To identify the sequence forming the transmembrane β-hairpins we carried out fluorescence spectroscopy studies using single cysteine mutants in TMH1, as previously performed on CDCs [20]. This approach revealed an alternating pattern of emission between residues 128–147 consistent with a ∼30 Å membrane-spanning amphipathic β-hairpin structure (S1 Fig.). This information provided a useful restraint for the fitting. In the resulting pore model, each MACPF domain forms a four-stranded β-sheet (Fig. 3A–3C).

β-barrels are limited to discrete architectures, each with a characteristic strand tilt relative to the barrel axis [31]. For a barrel composed of *n* strands, the shear number *S* describes the register of hydrogen bonding between residues in adjacent β-strands and defines the strand tilt and the dimensions of the formed barrel: the greater the strand tilt, the wider and shorter the barrel [32]. Only three Ply barrel models, with *S* = 0 (0° tilt), *S* = *n*/2 (20° tilt), and *S* = *n* (36° tilt) have dimensions comparable with the Ply pore cryo-EM map (S2 Fig.). The *S* = *n*/2 model gave the best fit in diameter and height (CC = 0.90 versus 0.73 for *S* = 0 barrel and 0.74 for *S* = *n*).

This 52-stranded β-barrel was combined with a 13-mer ring of fitted PlyB molecules. Because of steric clashes with the barrel, further refinement using Flex-EM was performed on the HTH motif (residues 298–313) (Figs. 1B and 3C, 3D). After refinement of the central asymmetric unit, the pore was rebuilt with C13 symmetry in Chimera [33] to give the final pore model. In this pore, the central β-sheet has straightened and opened by ∼70°, as measured from the fitting, and TMH1 and TMH2 are fully unwound into β-hairpins to form a β-barrel spanning the membrane bilayer (Fig. 3A–3C). The pore channel is thus formed by a 52-stranded β-barrel that is 80 Å in inner diameter and over 100 Å in height.

**Figure 4:**
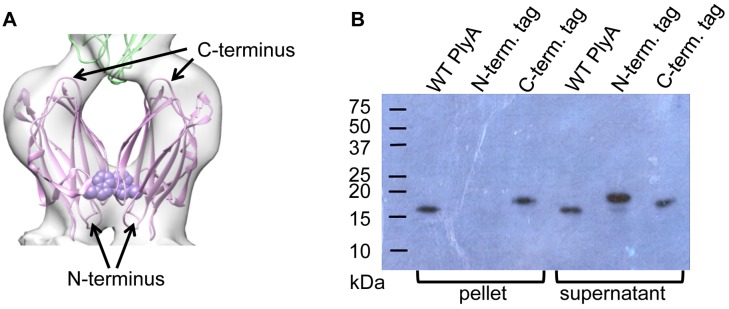
Validation of the orientation of PlyA. (A) Proposed orientation of PlyA dimer (pink) and interface with PlyB C-terminal trefoil (green). Trp 6 is shown as purple spheres. (B) Western blot showing PlyA binding to red blood cells when untagged or C-terminally tagged but not when N-terminally tagged.

The PlyB C-terminal trefoil sits in the cavity formed by a V-shaped wedge of density contacting the membrane (Figs. 3C and 4A). This density can be accounted for by two PlyA molecules, revealing a tridecameric PlyB/2xPlyA pore assembly. The symmetrical shape of PlyA precludes discrimination of up/down orientation in the density. However, in the crystal structure of PlyA, we noted two different V-shaped dimers (termed N-dimer and C-dimer) in the asymmetric unit (S3A and S3D Fig.). Both forms fitted adequately into EM density, placing either the PlyA N-terminus (N-dimer) or C-terminus (C-dimer) in proximity to the membrane surface. We tested the orientation of PlyA by adding a hexahistidine tag to the N-terminus (Fig. 4A and 4B), which abrogated membrane binding of PlyA to red blood cells whereas a C-terminal tag had no effect on binding (Fig. 4B). Also, mutation of Trp 6 (W6E), located in the PlyA N-dimer interface, reduced membrane binding and led to 100-fold lower pore-forming activity (Fig. 4A, denoted as purple spheres; S4A and S4B Fig.). These data support an N-dimer-like arrangement of PlyA molecules (Fig. 4A), consistent with the known orientation of actinoporins on the membrane surface [29].

The resulting fit of 26 PlyA and 13 PlyB subunits had a cross-correlation coefficient of 0.8 with the map which includes part of the membrane as measured in Chimera [33]. To evaluate the local quality of fit, the segment-based cross-correlation coefficient (SCCC) [34] was determined and plotted on the pore subunit structure (S5 Fig.). This analysis shows that the fit is more reliable for PlyB than for PlyA, because the map resolution is better in the region occupied by PlyB.

To probe the mechanism of pore assembly, we engineered a series of disulphide bonds to limit movement in either TMH1 or TMH2. As performed for perfringolysin O and other CDCs [35], the TMH regions were trapped by introducing cross-links to the central sheet or other adjacent regions in the monomer structure. This trapping allows oligomer assembly but prevents the TMH region from unfolding enough to insert into the membrane. The disulphide trap mutants were engineered on a background PlyB variant that lacks the wild type cysteine (C487A) in order to avoid incorrect disulphide bond formation. PlyB^C487A^ retains wild type activity according to haemolysis assay (S6A Fig.). We then determined the cryo-EM structures of three different prepore-locked variants.

### Structure of a TMH1 Trapped Intermediate

**Figure 5:**
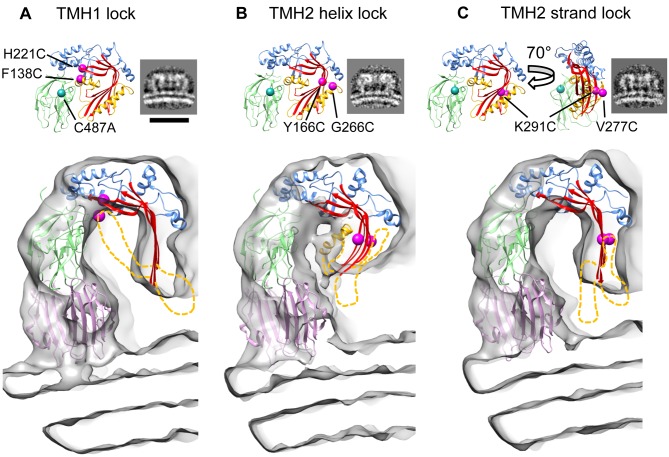
Three dimensional (3-D) reconstructions of disulphide locked pleurotolysin prepores. (A) PlyB crystal structure with positions of TMH1 disulphide lock indicated by magenta spheres and corresponding side view average of the liposome-bound prepore (cryo-EM). Scale bar, 20 nm. Main panel, cut-away view of the prepore cryo-EM map with the model obtained by flexible fitting. No TMH density is seen in the TMH1 lock prepore map. (B) The equivalent panels are shown for the TMH2 helix lock map and model. Partial density is seen for the TMH1 region. (C) Equivalent views of the TMH2 strand lock map and model. No density is seen for the TMH regions. These regions must therefore be disordered and they were omitted from the fits. The disordered regions are shown schematically as yellow dashed lines; the long TMH1 helix is illustrated in (B) but was not part of the fitting. Mutated residues are shown: TMH1 lock; F138C (located in the TMH1 region, yellow), H221C. TMH2 helix lock; Y166C, G266C (located in the TMH2 helix region, yellow). TMH2 strand lock; V277C (located in the fifth β-strand, TMH2 region, yellow), K291C, all on the C487A background.

Oxidised TMH1 variant PlyB^F138C,H221C^ (TMH1 lock) (Fig. 5A) possessed no detectable lytic activity (S6B Fig.), but reduction of the disulphide restored wild type lytic activity. Furthermore, the oxidised form could assemble into oligomeric prepores on erythrocytes or liposomes, and these prepores could be converted into lytic pores by disulphide reduction (S6C Fig.). These data suggest that the TMH1 lock prepore structure is an intermediate in the formation of the pore. The crystal structure of the TMH1 trapped variant was determined and is otherwise indistinguishable from the wild type (S7 Fig.; S3 Table).

In order to analyse the degree of β-sheet opening we created a library of thousands of molecular models and then performed constrained fitting into the prepore map. This procedure is described in S8 Fig. Briefly, using the monomer and pore structures as end points, we generated two series of angular sweeps of the beta sheet opening or closing, using the TEMPy software [36]. Each of these 2,300 generated β-sheet conformations was combined twice with the rest of the PlyB structure, either with the monomer or with the pore conformation, using MODELLER/Flex-EM [30]. The resulting 4,600 models were assessed by their fit to the prepore map and by statistical potentials, and the best ranking fits were used to estimate the angle of β-sheet opening in the prepore conformation (S4 Table).

Remarkably, the cryo-EM 3-D structure of the TMH1 lock prepore showed that the central sheet of the MACPF domain in the prepore assembly had opened substantially (53° ± 9°) (Fig. 5A). In these prepores, the top half of the barrel has formed, but not the lower, transmembrane part. Indeed, no density could be observed for most of TMH1 and TMH2 and it appears that these regions are largely unstructured. Thus, these data reveal that locking TMH1 has little effect in limiting the MACPF β-sheet opening.

### Structures of TMH2 Trapped Intermediates Identify a Key Region for Controlling Sheet Opening

To lock TMH2 to the central core β-sheet of the MACPF domain we identified a second variant PlyB^Y166C,G266C^ (TMH2 helix lock) that could be trapped in the prepore state (Fig. 5B). As with TMH1 lock, addition of reducing agent allows it to continue its trajectory to the pore form (S6D and S6E Fig.). The cryo-EM structure of the TMH2 helix lock prepore revealed a very different picture from the TMH1 lock. The core sheet opening is only 37° ± 11° with some density remaining for TMH1, suggesting that this region remains partly ordered (Fig. 5B). These data show that the MACPF fold oligomerises without substantial relief of the twist in the core sheet or TMH1 release. As in the TMH1 lock prepore, no density can be seen extending to the membrane surface, consistent with the absence of lytic function. The β-sheet opening was analysed as above (S8 Fig.). Remarkably, the results reveal that restricting the movement of the top of the TMH2 α-helix prevents unbending of the MACPF core β-sheet. This finding is opposite to expectation, since TMH1 forms an extensive buried interface, whereas TMH2 makes fewer contacts with the domain core sheet. However, our results are consistent with observations that TMH2 is important for controlling pore formation in other superfamily members, including interactions with the MAC inhibitor CD59, and the pH trigger of the CDC listeriolysin O [25,37,38].

To further probe TMH2 function, a third PlyB disulphide lock was created to join strands β4 of the central β-sheet to β5 of TMH2, PlyB^V277C,K291C^ (TMH2 strand lock; Figs. 5C, S6F, and S6G). Cryo-EM and modelling analysis showed that the central β-sheet was open to the same extent as in the TMH1 lock (Fig. 4C, 49° ± 8°). This result provides an interesting contrast to the consequences of the TMH2 helix lock and highlights that simply restricting TMH2 movement through locking strands β4 and β5 does not prevent opening of the core sheet. The restrictions enforced by the TMH2 strand lock are, however, sufficient to prevent extension of the TMH2 hairpin since no density is seen extending into the membrane. These data collectively imply that neither TMH region can enter the membrane without the other, suggesting that the TMH sequences have evolved for cooperative folding and assembly.

## Discussion

Here, we present a series of structures that identify the major conformational changes during MACPF pore formation. The final pore structure reveals that individual PlyB monomers in the pore have the orientation seen for those in the distantly related CDCs [17]. Although sequence-related to perforin, their pores differ in several respects. Like CDCs, perforin is a thin, key-shaped molecule, but it does not open up in the pore state [18]. This difference likely arises from the divergent structures surrounding the conserved MACPF core, as well as from its longer TMH regions. In addition, C-terminal labelling indicated the opposite β-sheet orientation in the perforin pore [18]. A model based on a more recently determined C8 structure [39] suggests that the closely related terminal complement proteins would have the CDC orientation, but there are currently no other data available for a more definitive conclusion on perforin.

Our findings highlight a critical role of the interface between the top of TMH2 and the surrounding region in controlling sheet opening. The results of the constrained fitting suggest that a key trigger for the conformational change includes displacement of the HTH motif away from the bend in the sheet. Highly conserved glycine residues [14] adjacent to the HTH motif may provide the hinge point for this motion (Fig. 3D). Consistent with this model, mutation of the equivalent glycine residues in a CDC prevents oligomerisation [40]. It is notable that the HTH packs against the top of TMH2, suggesting that interactions between these two regions may govern unlocking of the bent conformation (Fig. 3D). After sheet unbending, we propose that membrane insertion and pore formation follow a top down, zippering mechanism with the barrel assembling towards the membrane surface, energetically driven by refolding of the TMH regions. This mechanism would also minimize the free energy cost of inserting naked hairpins with unsatisfied hydrogen bond potential into the membrane.

**Figure 6:**
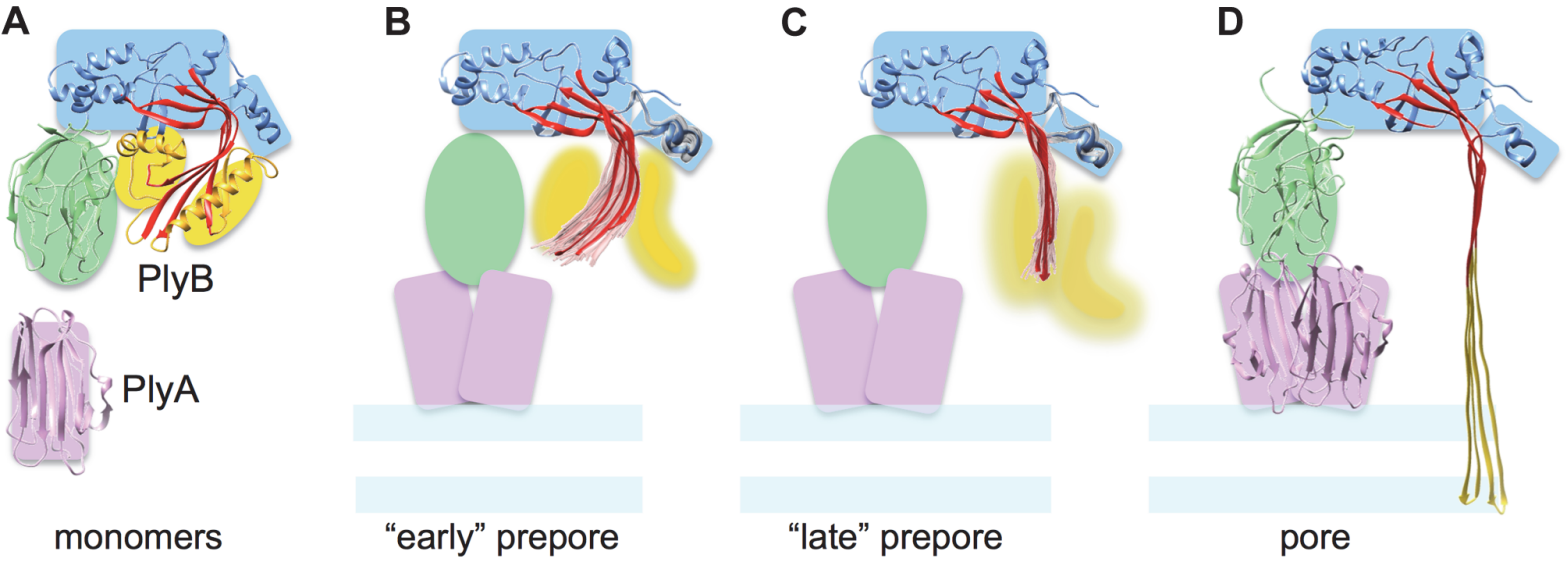
Schematic diagram of sheet opening in pleurotolysin pore formation. (A) PlyA and B monomer structures with coloured shapes indicating the major elements used in flexible fitting. (B) and (C) show the two types of prepore structures found, which can be considered as early and later stages of sheet opening. The top 20 models (pink for the MACPF β-sheet and grey for HTH region) and best scoring model (red) in the pleurotolysin prepore maps are shown. (B) TMH2 helix lock, in which the best scoring model has a rotation angle (β-sheet opening) of 37° relative to the monomer structure. (C) TMH2 strand lock, in which the best scoring model has a rotation angle of 49° relative to the monomer. (D) Pore model with 70° open sheet and membrane inserted TMH regions.

Analysis of intermediate prepore structures provides the basis for a molecular movie (S1 Movie) that illustrates a possible trajectory of the core β-sheet in a MACPF protein unbending from the soluble monomer conformation to the transmembrane pore (Fig. 6). The pore structure shows that Ply shares some features with CDCs, in particular the orientation of monomers and opening of the molecule to release the TMH regions. On the other hand it resembles perforin regarding its longer TMH regions that refold into a ∼100-Å-long beta-barrel that reaches down through the membrane without any collapse of the molecule.

This work provides new insights into the assembly of a two-component MACPF/CDC family member, suggesting a basis for the study of more complex assembly systems such as the complement MAC. Furthermore, the intermediate structures of the MACPF/CDC domain during its refolding into the β-barrel pore establish a structural paradigm for the transition of the prepore to pore, which is likely to be conserved across the MACPF/CDC protein family.

## Materials and Methods

### Protein Production and Crystallography

PlyA and PlyB were expressed in *Escherichia coli*. PlyA was expressed as a soluble protein; PlyB required refolding. Crystals of selenomethionine-labelled PlyA were grown in 50 mM Na citrate (pH 5.6), 12% (w/v) PEG3350, 0.2 M MgSO_4_, and the structure determined using single-wavelength anomalous dispersion (S1 Table). Crystals of PlyB were grown in 0.2 M NH_4_Ac, 0.1 M Na citrate (pH 5.0) and 30% (w/v) PEG8000. In addition to two native datasets, diffraction data were also collected from four different heavy atom derivatives (S2 Table). The PlyB structure was determined using multiple isomorphous replacement with anomalous scattering. The PlyB TMH1 lock structure was determined by molecular replacement using the structure of wild type PlyB.

### Electron Microscopy and Atomic Structure Fitting

Pleurotolysin WT pores and engineered disulphide bond oligomers assembled on sphingomyelin/cholesterol liposomes were imaged by negative stain and cryo-EM, and sorted according to diameter and symmetry by multivariate statistical analysis and multireference alignment. Three-dimensional reconstructions were obtained by a combination of angular reconstitution and projection matching for tridecameric pores and prepores. The PlyA and PlyB crystal structures and a model for a 52-stranded β-barrel [31] were fitted into the electron microscopy maps using a combination of manual fitting, rigid body refinement, and flexible fitting. The degree of unbending of the MACPF β-sheet in the prepore intermediates was estimated by using a multistep procedure (S8 Fig.) to generate a library of sheet conformations in angular sweeps, and selecting the top 20 best fitting ones for each prepore map. The goodness-of-fit of the central asymmetric unit was assessed locally using SCCC (S5 Fig.) [33,34].

Detailed protocols are available in S1 Methods.

## Supporting Information

S1 Fig TMH1 unravels to form a membrane penetrating amphipathic β-hairpin. (A) The fluorescence emission of NBD covalently attached to the individual cysteine substituted residues along the putative TMH1 hairpin is shown in the absence and presence of cholesterol-rich liposomes. Dark grey, residues in a hydrophobic environment; light grey, residues facing the barrel lumen; black bars, proposed β-hairpin residues; and the asterisks denote mutant proteins that could not be produced in functional form. (B) Collisional quenching of the NBD probe by a C12 doxyl group positioned on the acyl chain of phosphatidylcholine. The fluorescence data from TMH1 show an identical pattern to that observed previously for both membrane-inserted TMHs of perfringolysin O where two residues immediately prior to the β-turn are membrane associated and then the alternating pattern of membrane inserted residues is offset by one residue in the second strand [41]. The position of proline residue 137 in TMH1 is consistent with the suggested position of the β-turn. (C) Schematic of the TMH1 β-hairpin with strand 1, predicted hairpin, then strand 2. Orange boxes are residues that are in contact with the membrane, blue boxes are residues facing the lumen of the barrel, black boxes are the β-turn residues, and white boxes represent mutant proteins that could not be produced in functional form.
(TIF)

S2 Fig Fitting of different β-barrel models into the pleurotolysin pore reconstruction. The diameter and the height of β-barrels depend on the number of strands (*n*) and shear (*S*) of the β-hairpins relative to the barrel axis. (A) *S* = 0 (0° tilt) corresponds to a β-barrel with strands parallel to the barrel axis. (B) *S* = *n*/2 (20° tilt) model. For a pleurotolysin pore with 13-fold symmetry and each subunit contributing two hairpins the shear *S* is 26 [31]. (C) *S* = *n* (36° tilt) and higher correspond to greater tilts of the β-strands and are typically associated with small transmembrane β-barrels. (D) Fitting of pleurotolysin β-barrel models into the cryo-EM map of the pleurotolysin pore revealed that the barrel height in models with *S* = *n* (purple) and higher shear is not sufficient to cross the membrane. Of the three possible models, *S* = *n*/2 (blue) fits significantly better (cross-correlation 0.90) than *S* = 0 (green; cross-correlation 0.73) and *S* = *n* (purple; cross-correlation 0.74). Coloured bars indicate the bottom of each corresponding barrel fitted in the pore map.
(TIF)

S3 Fig Comparison of PlyA orientation with actinoporins and domain 4 in CDCs. Two crystal forms of PlyA revealed two different V-shaped dimers. (A) An N-terminus-N-terminus dimer (N-dimer) of PlyA, which when fitted into EM density is positioned on the membrane similarly to actinoporins [29]; (B) Sticholysin II [29], phosphocholine is shown in space filling format (grey spheres). (C) Superposition of PlyA and sticholysin II structures. (D) C-terminus-C-terminus dimer (C-dimer) of PlyA, which fits into EM density in the orientation corresponding to that of the C2 domain of perforin [18]. (E) Domain 4 of perfringolysin O [42] with the cholesterol binding residues shown as cyan spheres. Both PlyA dimers (A and D) could be fitted into the pleurotolysin pore density, with a slightly better fit for the N-dimer (cross-correlation 0.74 versus 0.71 for C-dimer).
(TIF)

S4 Fig Effect of His tags on the binding of PlyA to membranes and haemolysis. To test the subunit orientation in the fit, we mutated a residue (W6E) located in the centre of the PlyA N-N dimer interface (Fig. 4A). (A) Western blot analysis of a red blood cell (RBC) pull-down assay by PlyA. This assay shows a reduced affinity of W6E PlyA compared to WT PlyA. (B) The W6E PlyA variant has significantly reduced (90–120-fold, duplicate experiment) pleurotolysin lytic activity.
(TIF)

S5 Fig Local assessment of the quality of fit for a pore subunit. The local quality of fit in each region (defined as in Fig. 1) was assessed using SCCC [34]. The pseudo-atomic pore subunit model is shown colour-coded according to the SCCC score from blue to red (see colour key).
(TIF)

S6 Fig Concentration and time dependence of haemolysis activity of wild type and mutant pleurotolysin. (A) Titration based haemolysis assay of wild type pleurotolysin and PlyB^C487A^ mutant. Titration (B) and time course haemolysis assays (C) of TMH1 lock mutant, TMH2 helix lock mutant (D, E) and TMH2 strand lock mutant (F, G). In (B), (D), (F) all disulphide locked mutants are compared to the PlyB^C487A^ background in reducing and non-reducing conditions. All the locked mutants lack haemolytic activity in non-reducing conditions (empty squares) compared to reducing conditions (black squares). Titration assay shows that reduced TMH1 lock (B) and TMH2 strand lock (F) mutants recovered the same activity as the background mutant, whereas the TMH2 helix lock (D) recovered 50% lysis activity at 3-fold higher concentration than the background mutant. In the time-course haemolysis assay of prepores (C, E, G), red blood cells were incubated with disulphide locked mutants to form prepores on the surface of the cells. Unbound PlyA and PlyB were then washed off. Conversion of prepores to pores was monitored (at 620 nm) in real time by decrease of light scattering resulting from lysis of the cells (black circles), compared to either pre-lysed cells (empty triangles) or prepore loaded RBC in non-reducing conditions (empty circles). Note that due to the long time required to lyse the cells there is settling of the RBC in the negative control sample. These data show that all assembled pleurotolysin prepores can convert to pores under reducing conditions.
(TIF)

S7 Fig Overlay of PlyB crystal structure with PlyB^F138C,H221C^ (TMH1 lock, purple). RMSD 0.47 Å (463 Cα). The disulphide bond is shown as sticks, with the 2*F*_*o*_-*F*_*c*_ electron density contoured at 1*σ* level shown in blue (inset).
(TIF)

S8 Fig Flowchart of PlyB conformational analysis for the three prepore maps. The input models (A) correspond to the PlyB crystal structure and the pore subunit model (based on density fitting of PlyA and PlyB crystal structures and the β-barrel into the pore density map). These were used as initial conformations for two series of angular sweeps of the MACPF β-sheet (B), generated in steps of 0.5 Å translation and 1° rotation around the centre of mass. Each sampled conformation of the MACPF β-sheet was then combined with the PlyB monomer structure or with the pore model, resulting in a library of ∼4,600 models (C). Each model was then rigidly fitted into each of the prepore maps (D) and the goodness-of-fit of the MACPF β-sheet was assessed using SCCC [34]. The top 20 models were then re-ranked (E) based on the DOPE score [43].
(TIF)

S9 Fig Resolution curves for cryo-EM reconstructions. (TIF)

S1 Movie Movie of the trajectory from soluble monomers (crystal structures) through to the final assembled pore shows the opening of the β-sheet. The cryo-EM reconstruction of the first intermediate (TMH2 helix lock) is shown with the model. This is followed by the cryo-EM reconstruction of the TMH1 lock with the best-fit model. The final images show the 11 Å resolution cryo-EM reconstruction of the assembled MACPF pore.
(MOV)

S1 Methods Detailed protocols not described in the Materials and Methods section. (DOCX)

S1 Table Data collection and statistics for PlyA crystals. (DOCX)

S2 Table Data collection and statistics for PlyB crystals. (DOCX)

S3 Table Data collection and statistics for PlyB ^C487A,F138C,H221C^ crystals. (DOCX)

S4 Table Domain-orientation score [30] (DOS, translation, and rotation parameters) for 20 best fitting sheet conformations for TMH2 helix lock, TMH1 lock, and TMH2 strand lock pleurotolysin prepores (Fig. 6). (DOCX)

## Abbreviations:

CDC: cholesterol dependent cytolysin
EM: electron microscopy
HTH: helix-turn-helix
MACPF: membrane attack complex/perforin-like family
SCCC: segment-based cross-correlation score
TMH: transmembrane hairpin
