# Supplementary figures and images for: Conformational Changes during Pore Formation by the Perforin-Related Protein Pleurotolysin
Source: PLoS Biol. 2015 Feb 5;13(2):e1002049. doi: 10.1371/journal.pbio.1002049 (PMC4318580; doi:10.1371/journal.pbio.1002049)

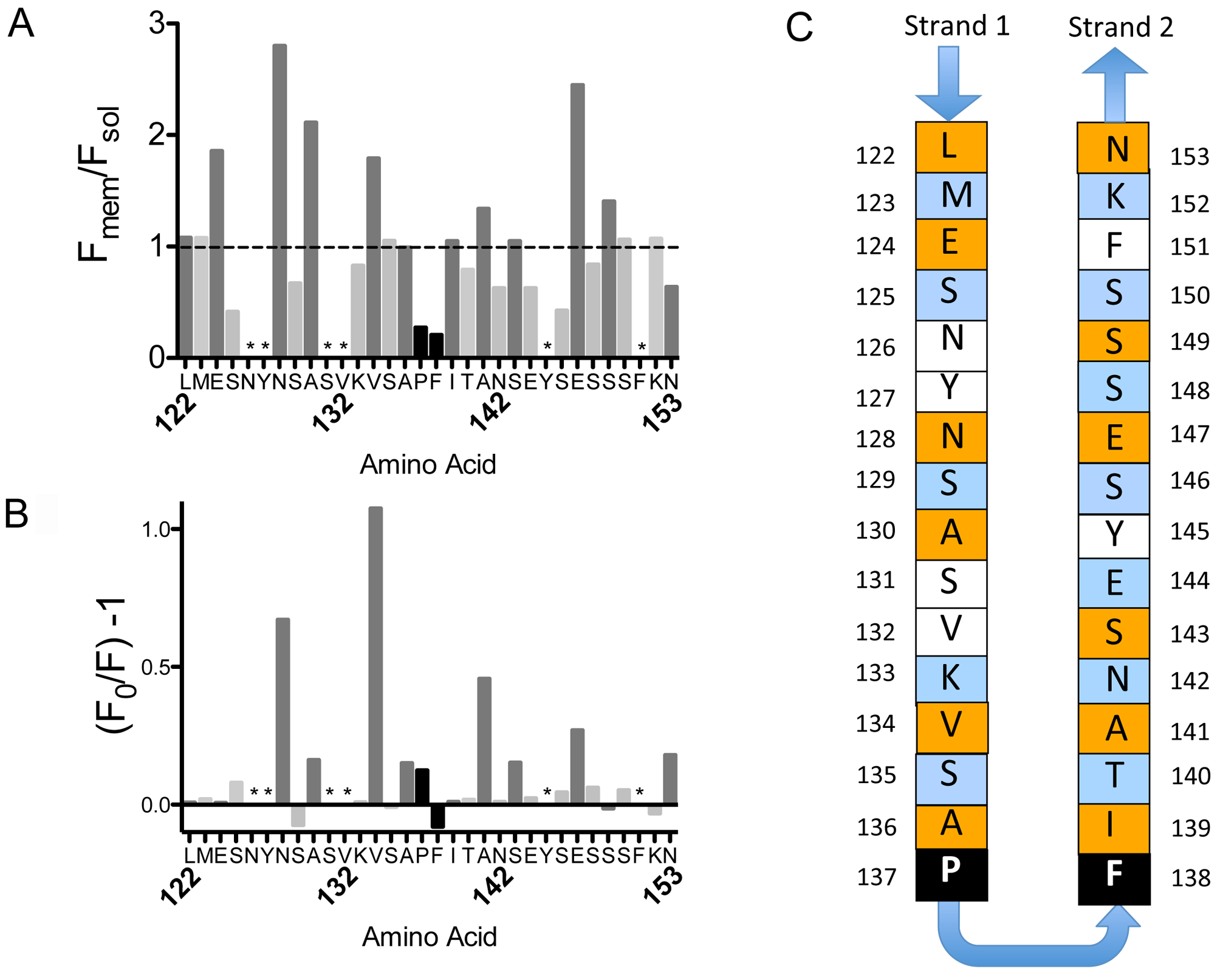

Supplement: S1 Fig — (A) The fluorescence emission of NBD covalently attached to the individual cysteine substituted residues along the putative TMH1 hairpin is shown in the absence and presence of cholesterol-rich liposomes. Dark grey, residues in a hydrophobic environment; light grey, residues facing the barrel lumen; black bars, proposed β-hairpin residues; and the asterisks denote mutant proteins that could not be produced in functional form. (B) Collisional quenching of the NBD probe by a C12 doxyl group positioned on the acyl chain of phosphatidylcholine. The fluorescence data from TMH1 show an identical pattern to that observed previously for both membrane-inserted TMHs of perfringolysin O where two residues immediately prior to the β-turn are membrane associated and then the alternating pattern of membrane inserted residues is offset by one residue in the second strand [41]. The position of proline residue 137 in TMH1 is consistent with the suggested position of the β-turn. (C) Schematic of the TMH1 β-hairpin with strand 1, predicted hairpin, then strand 2. Orange boxes are residues that are in contact with the membrane, blue boxes are residues facing the lumen of the barrel, black boxes are the β-turn residues, and white boxes represent mutant proteins that could not be produced in functional form. (TIF) [file pbio.1002049.s001.tif]

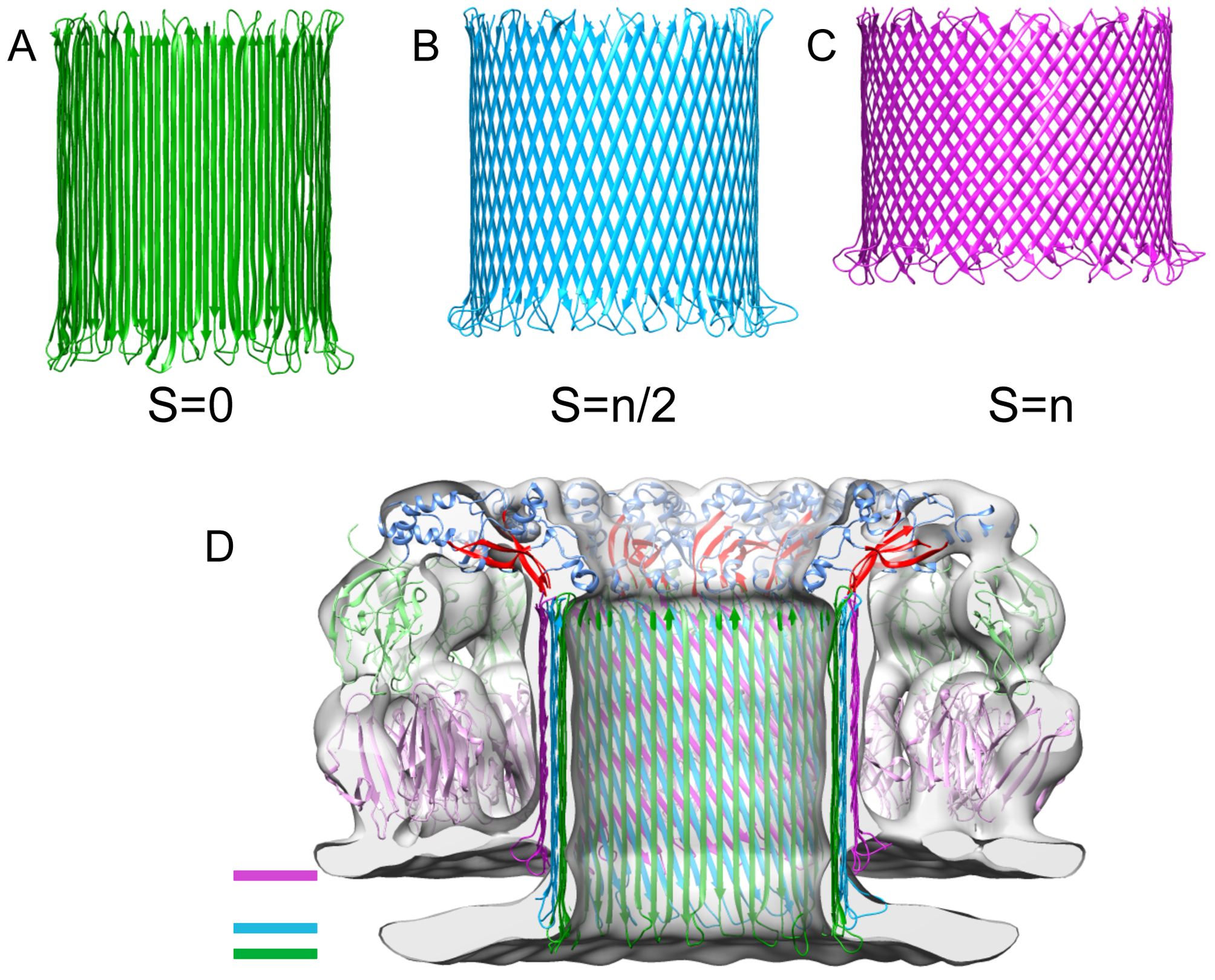

Supplement: S2 Fig — The diameter and the height of β-barrels depend on the number of strands (n) and shear (S) of the β-hairpins relative to the barrel axis. (A) S = 0 (0° tilt) corresponds to a β-barrel with strands parallel to the barrel axis. (B) S = n/2 (20° tilt) model. For a pleurotolysin pore with 13-fold symmetry and each subunit contributing two hairpins the shear S is 26 [31]. (C) S = n (36° tilt) and higher correspond to greater tilts of the β-strands and are typically associated with small transmembrane β-barrels. (D) Fitting of pleurotolysin β-barrel models into the cryo-EM map of the pleurotolysin pore revealed that the barrel height in models with S = n (purple) and higher shear is not sufficient to cross the membrane. Of the three possible models, S = n/2 (blue) fits significantly better (cross-correlation 0.90) than S = 0 (green; cross-correlation 0.73) and S = n (purple; cross-correlation 0.74). Coloured bars indicate the bottom of each corresponding barrel fitted in the pore map. (TIF) [file pbio.1002049.s002.tif]

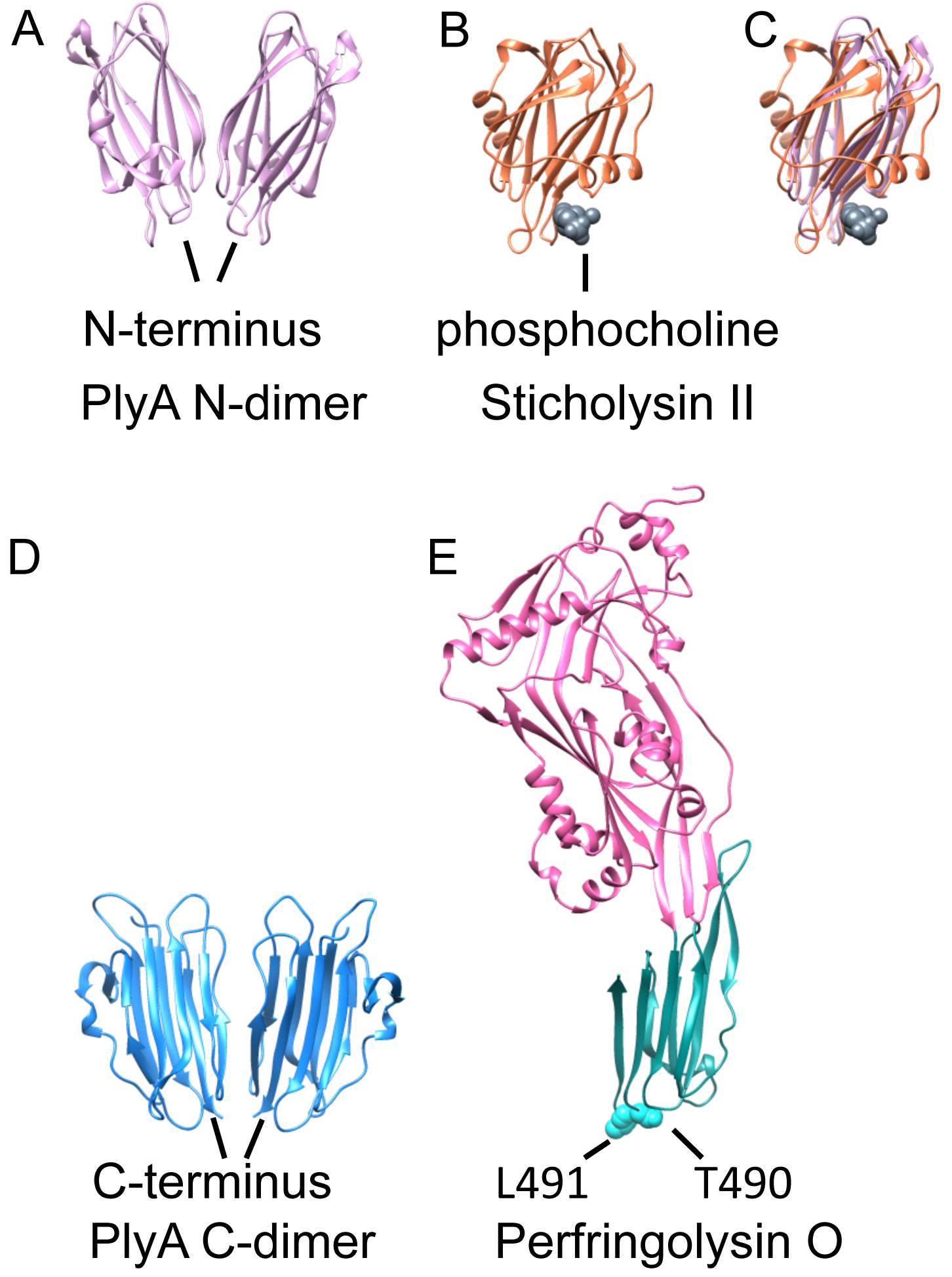

Supplement: S3 Fig — Two crystal forms of PlyA revealed two different V-shaped dimers. (A) An N-terminus-N-terminus dimer (N-dimer) of PlyA, which when fitted into EM density is positioned on the membrane similarly to actinoporins [29]; (B) Sticholysin II [29], phosphocholine is shown in space filling format (grey spheres). (C) Superposition of PlyA and sticholysin II structures. (D) C-terminus-C-terminus dimer (C-dimer) of PlyA, which fits into EM density in the orientation corresponding to that of the C2 domain of perforin [18]. (E) Domain 4 of perfringolysin O [42] with the cholesterol binding residues shown as cyan spheres. Both PlyA dimers (A and D) could be fitted into the pleurotolysin pore density, with a slightly better fit for the N-dimer (cross-correlation 0.74 versus 0.71 for C-dimer). (TIF) [file pbio.1002049.s003.tif]

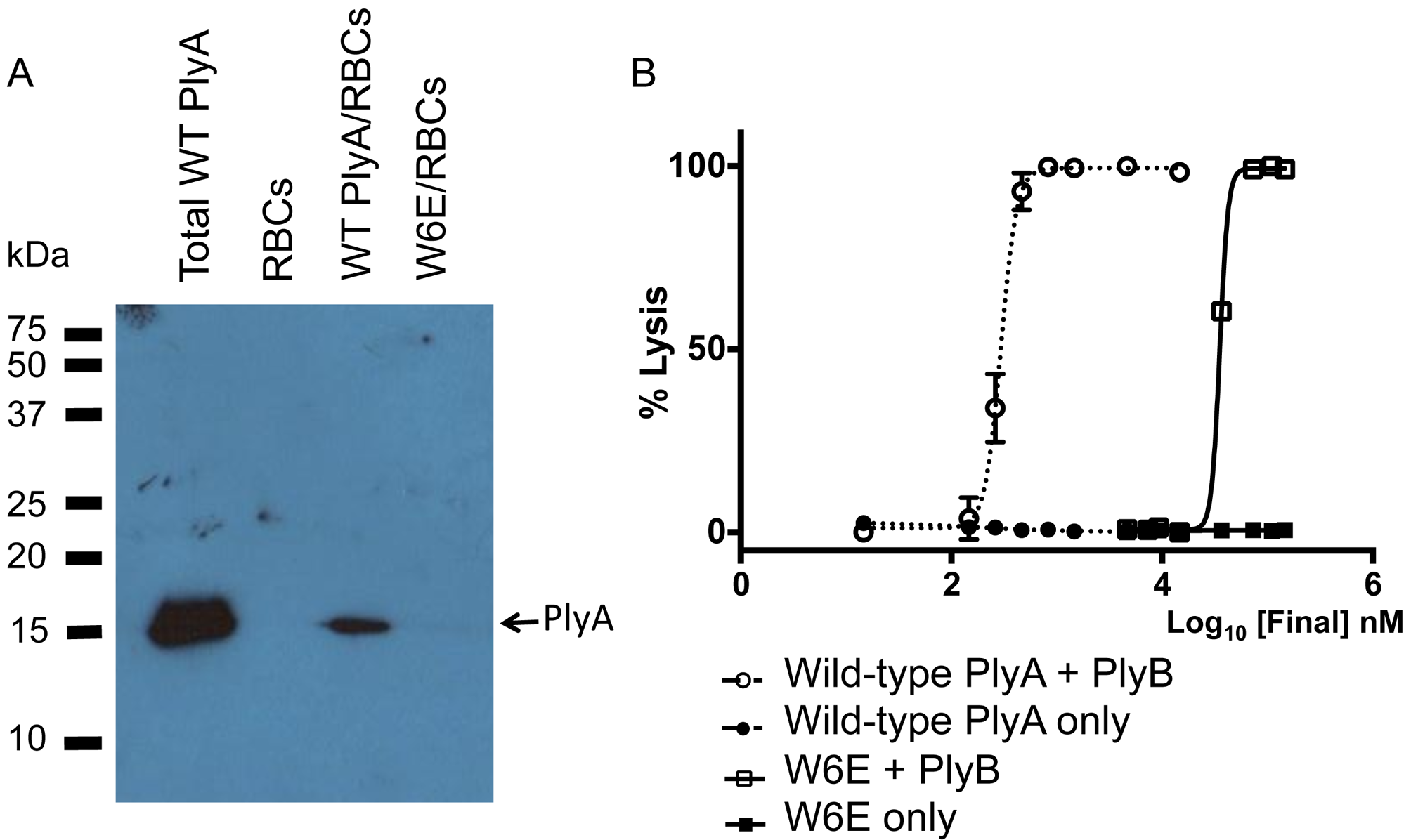

Supplement: S4 Fig — To test the subunit orientation in the fit, we mutated a residue (W6E) located in the centre of the PlyA N-N dimer interface (Fig. 4A). (A) Western blot analysis of a red blood cell (RBC) pull-down assay by PlyA. This assay shows a reduced affinity of W6E PlyA compared to WT PlyA. (B) The W6E PlyA variant has significantly reduced (90–120-fold, duplicate experiment) pleurotolysin lytic activity. (TIF) [file pbio.1002049.s004.tif]

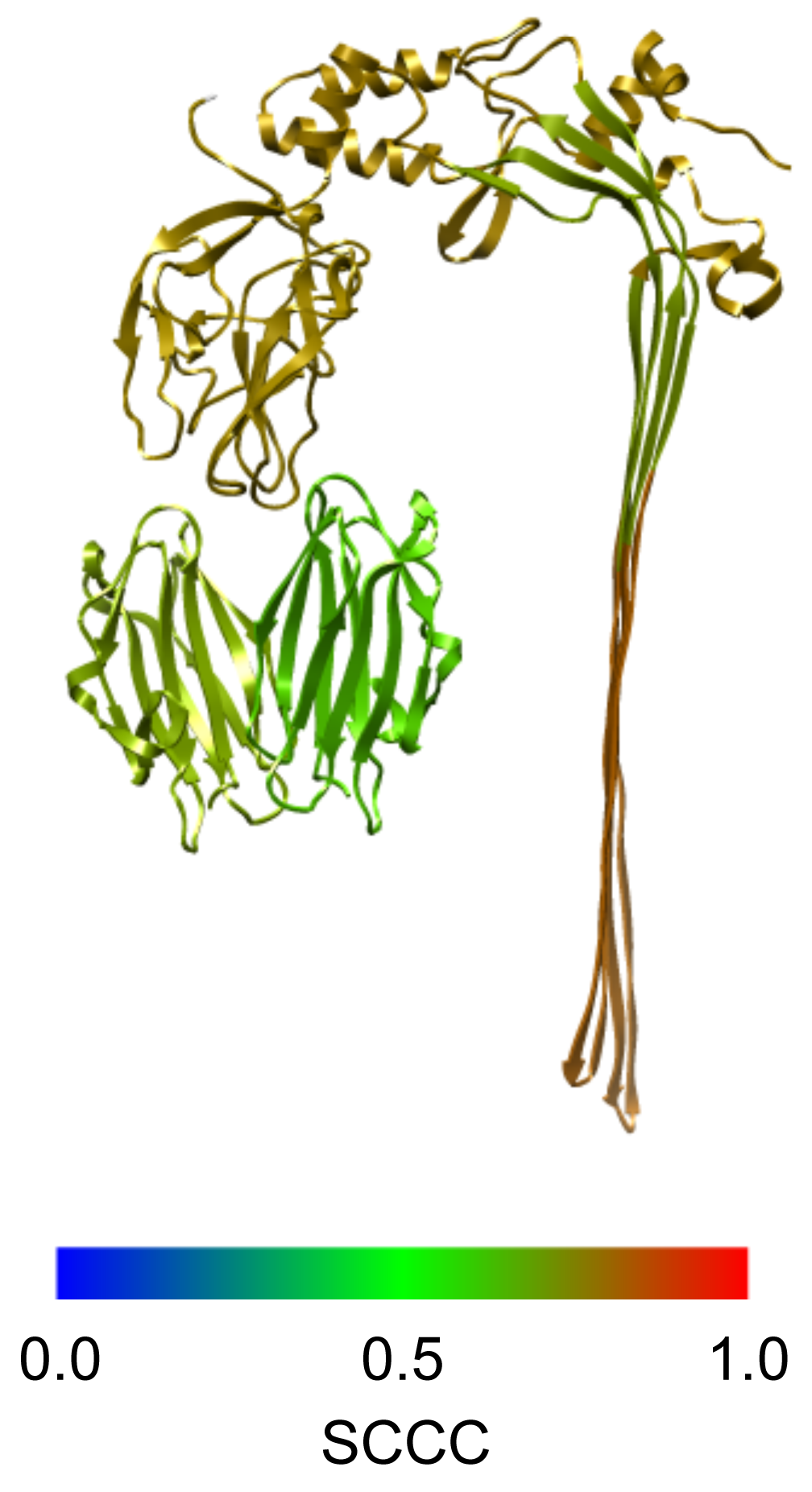

Supplement: S5 Fig — The local quality of fit in each region (defined as in Fig. 1) was assessed using SCCC [34]. The pseudo-atomic pore subunit model is shown colour-coded according to the SCCC score from blue to red (see colour key). (TIF) [file pbio.1002049.s005.tif]

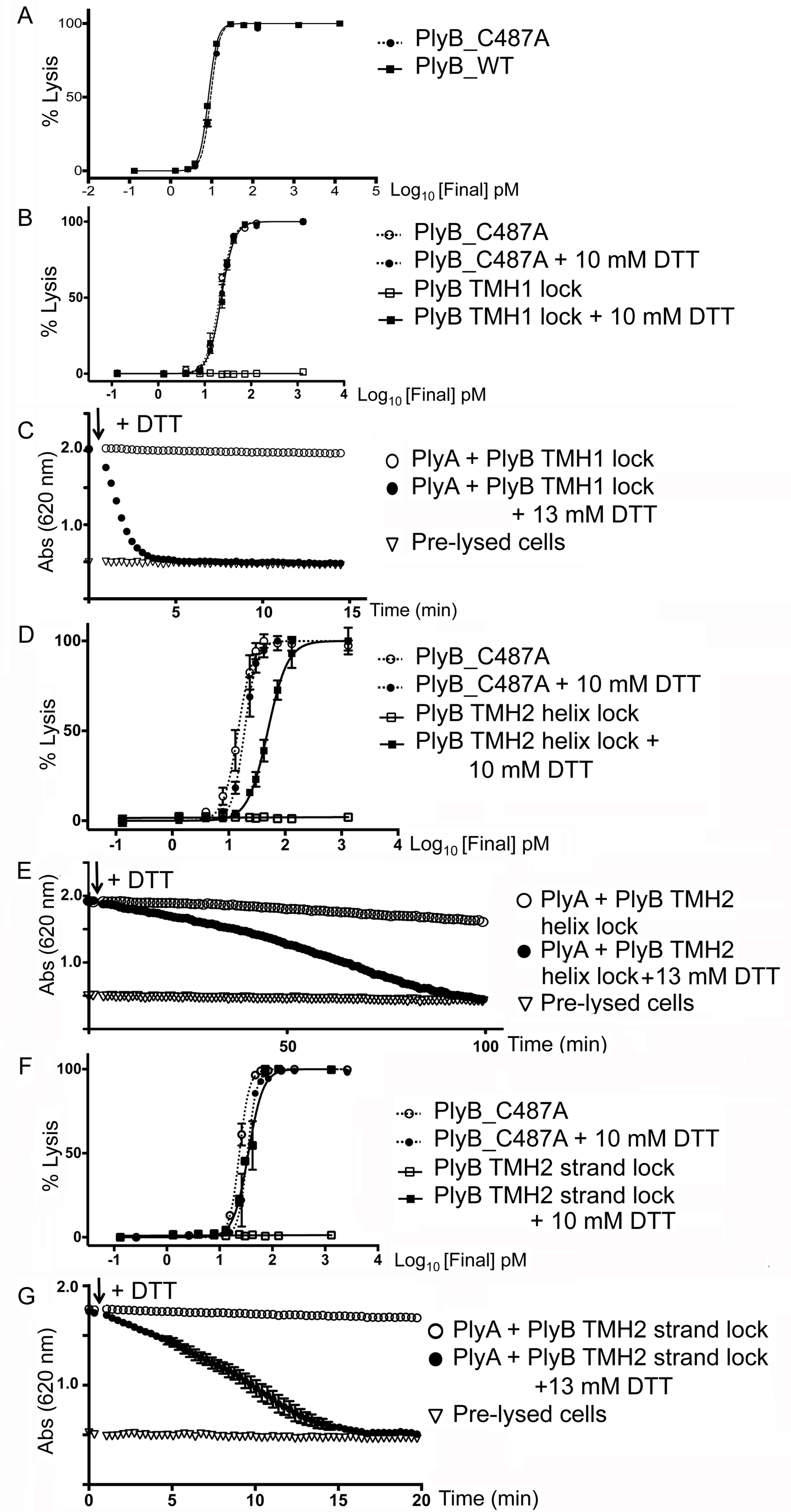

Supplement: S6 Fig — (A) Titration based haemolysis assay of wild type pleurotolysin and PlyBC487A mutant. Titration (B) and time course haemolysis assays (C) of TMH1 lock mutant, TMH2 helix lock mutant (D, E) and TMH2 strand lock mutant (F, G). In (B), (D), (F) all disulphide locked mutants are compared to the PlyBC487A background in reducing and non-reducing conditions. All the locked mutants lack haemolytic activity in non-reducing conditions (empty squares) compared to reducing conditions (black squares). Titration assay shows that reduced TMH1 lock (B) and TMH2 strand lock (F) mutants recovered the same activity as the background mutant, whereas the TMH2 helix lock (D) recovered 50% lysis activity at 3-fold higher concentration than the background mutant. In the time-course haemolysis assay of prepores (C, E, G), red blood cells were incubated with disulphide locked mutants to form prepores on the surface of the cells. Unbound PlyA and PlyB were then washed off. Conversion of prepores to pores was monitored (at 620 nm) in real time by decrease of light scattering resulting from lysis of the cells (black circles), compared to either pre-lysed cells (empty triangles) or prepore loaded RBC in non-reducing conditions (empty circles). Note that due to the long time required to lyse the cells there is settling of the RBC in the negative control sample. These data show that all assembled pleurotolysin prepores can convert to pores under reducing conditions. (TIF) [file pbio.1002049.s006.tif]

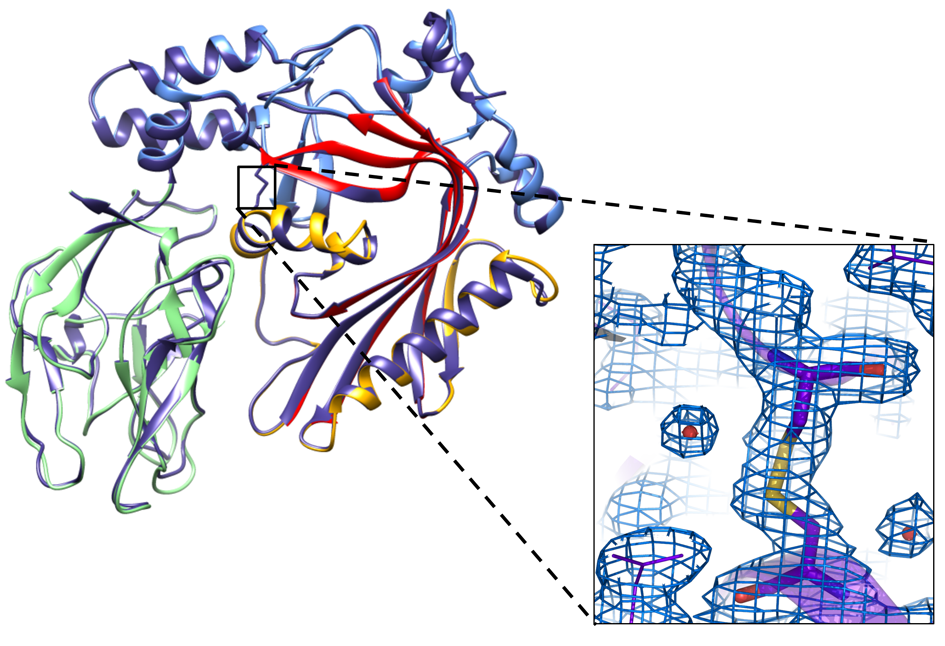

Supplement: S7 Fig — RMSD 0.47 Å (463 Cα). The disulphide bond is shown as sticks, with the 2F o-F c electron density contoured at 1σ level shown in blue (inset). (TIF) [file pbio.1002049.s007.tif]

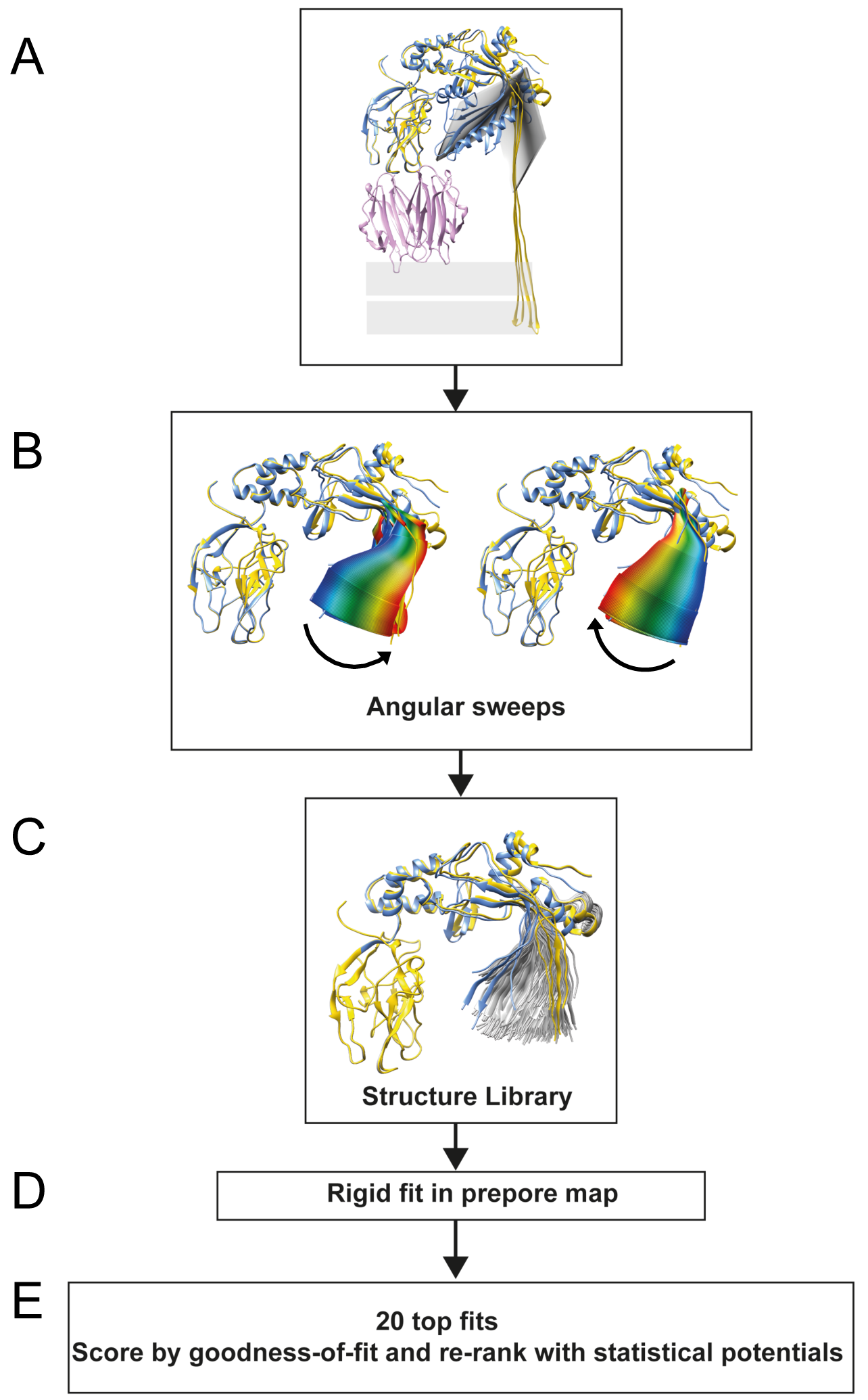

Supplement: S8 Fig — The input models (A) correspond to the PlyB crystal structure and the pore subunit model (based on density fitting of PlyA and PlyB crystal structures and the β-barrel into the pore density map). These were used as initial conformations for two series of angular sweeps of the MACPF β-sheet (B), generated in steps of 0.5 Å translation and 1° rotation around the centre of mass. Each sampled conformation of the MACPF β-sheet was then combined with the PlyB monomer structure or with the pore model, resulting in a library of ∼4,600 models (C). Each model was then rigidly fitted into each of the prepore maps (D) and the goodness-of-fit of the MACPF β-sheet was assessed using SCCC [34]. The top 20 models were then re-ranked (E) based on the DOPE score [43]. (TIF) [file pbio.1002049.s008.tif]

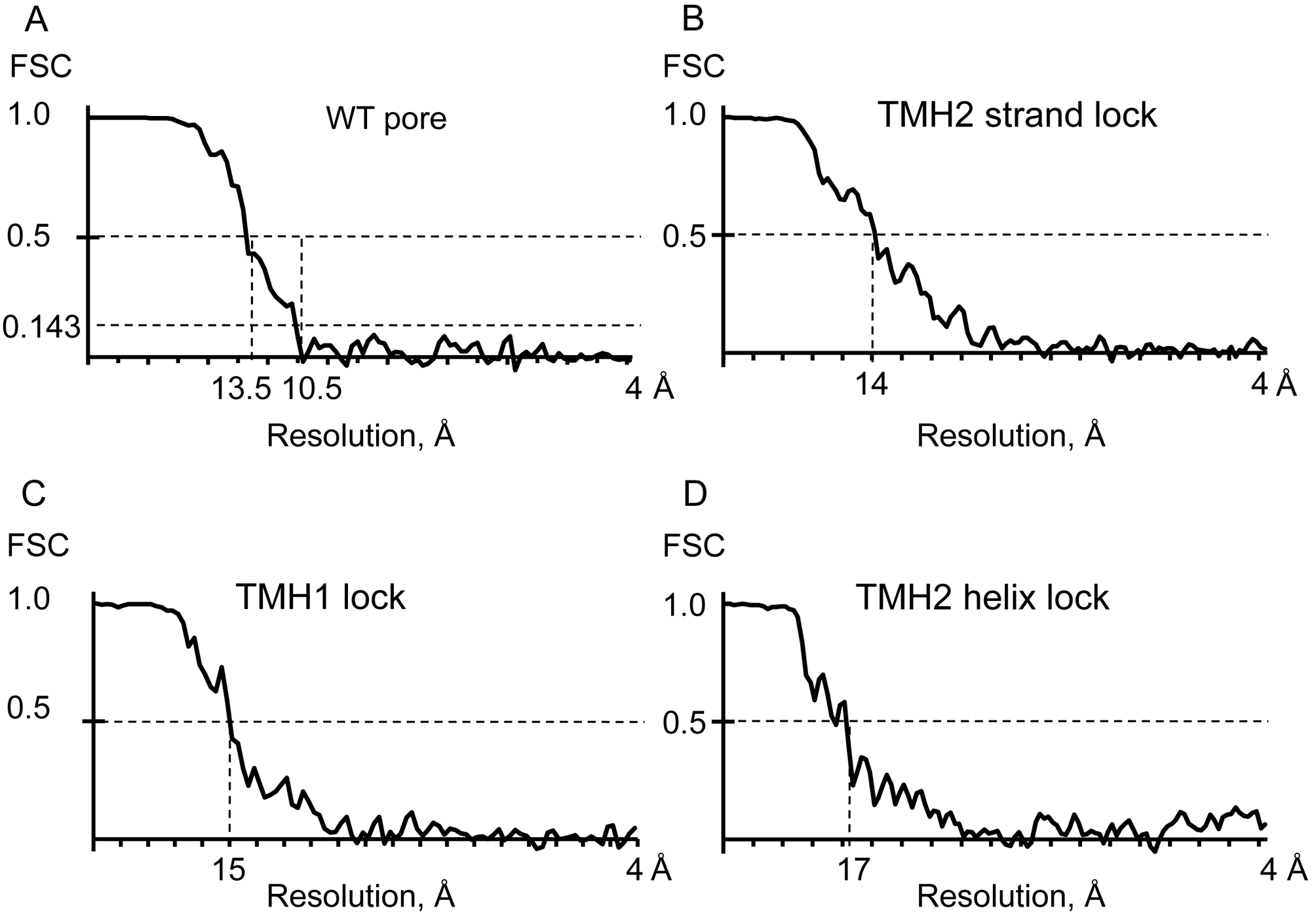

Supplement: S9 Fig — (TIF) [file pbio.1002049.s009.tif]
