# Supplementary material for: Conformational Changes during Pore Formation by the Perforin-Related Protein Pleurotolysin
Source: PLoS Biol. 2015 Feb 5;13(2):e1002049. doi: 10.1371/journal.pbio.1002049 (PMC4318580; doi:10.1371/journal.pbio.1002049)
Supplement: S1 Methods — (DOCX) [file pbio.1002049.s011.docx]

Methods S1

**PlyA Molecular Cloning and Expression**

The full-length PlyA gene was chemically synthesized (TopGene Technology), inserted into pUC57 (5’*Nde*I and 3’*Bgl*II). PlyA was then subcloned into pKN12 (N-terminal tagged PlyA) or into pET3a (tagless PlyA and C-terminal tagged PlyA). All plasmids were transformed into *E. coli* expression host cells BL21 Codon Plus (DE3) pLysS (Novagen).

For PlyA (all forms) the cells were grown in 500 ml 2 x YT media containing 100 mg ml^-1^ ampicillin and 34 mg ml^-1^ chloramphenicol for 2.5 h at 37°C before induction with 1 mM isopropyl β-D-1-thiogalactopyranoside (IPTG). The cell culture was then allowed to cool on the bench for 20 min before being grown for a further 20 h at 16°C, after which cells were harvested by centrifugation (3,000*g*, 4°C, 20 min). Cells containing PlyA were resuspended in 10 mM Tris-HCl pH 8.4, 10 mM EDTA. The cells were lysed by sonication and the soluble PlyA separated from the cell debris by centrifugation (15,000*g*, 4°C, 30 min).

Selenomethionine labeled PlyA (tagless form) was produced in a similar fashion using conditions adapted from Van Duyne *et al.* [[1](#_ENREF_1)]. Selenomethionine PlyA was purified as for native PlyA (see below) with the exception that all buffers contained freshly added 10 mM β-mercaptoethanol.

**PlyA purification**

For PlyA (tagless) purification, ammonium sulfate was added to the lysed cell supernatant, to a final concentration of 1.4 M at pH 8.4. The PlyA solution was centrifuged (15,000g, 4°C, 30 min) and the pellet containing PlyA precipitate was dissolved in 10 mM Tris-HCl pH 8.4. Undissolved material was removed by centrifugation (15,000*g*, 4°C, 15 min) and the supernatant was dialysed against 1 L of 10 mM Tris-HCl pH 8.4 buffer for 24 h with three buffer changes (1 L each dialysis). Dialysed material was centrifuged (15,000g, 4°C, 1 h) and supernatant was loaded onto a Q FF column (5 ml HiTrap, GE Healthcare) pre-equilibrated with 10 mM Tris-HCl pH 8.4. Protein was eluted with a linear gradient of 0.0–1 M KCl in the same buffer (12 column volumes, CV). Fractions containing the target protein were pooled and ammonium sulfate was added to a final concentration of 1.4 M. Solution was filtered using a 0.2 μm syringe filter (Pall) before being further purified using a phenyl-sepharose FF column (HiTrap, GE Healthcare) pre-equilibrated with 10 mM Tris-HCl pH 8.4 buffer with 1.4 M ammonium sulfate. Protein was eluted with a linear gradient of 1.4 – 0.0 M ammonium sulfate and fractions containing the target protein were pooled and further purified with size-exclusion chromatography (Superdex 75 16/60, GE Healthcare) pre-equilibrated with 10 mM Tris-HCl pH 8.4 and 200 mM KCl buffer.

For purification of N and C-terminally tagged PlyA the supernatant containing His-tagged PlyA was bound to a 1 mL HisTrap (GE Healthcare) column. The bound protein was washed with 2 CV of 300 mM NaCl, 10 mM imidazole, 0.01% (w/v) Triton X-100, 50 mM Tris pH 8.0 followed by 15 CV of 500 mM NaCl, 40 mM imidazole, 0.01% (w/v) Triton x-100, 50 mM Tris pH 8.0. Protein was eluted with a linear gradient of 0–300 mM imidazole in 20 CV in 150 mM NaCl, 50 mM Tris pH 8.0, 5% (v/v) glycerol and further purified using a Superdex 75 gel-filtration column (GE Healthcare) pre-equilibrated with 50 mM Tris pH 8.5, 150 mM NaCl, 1 mM EDTA, 5% (v/v) glycerol.

**PlyB Molecular Cloning**

The full-length PlyB gene was chemically synthesized (TopGene Technology) and inserted into a pUC57-derived vector. The mature fragment of the PlyB [[2](#_ENREF_2)] was sublconed cloned into either the pKN12 expression vector [[3](#_ENREF_3)] for expression of recombinant mature N-terminal His-tagged (His6-mPlyB) PlyB or pET3a vector for tagless PlyB (termed PlyB). His6-mPlyB contains a Tobacco Etch Virus (TEV) cleavage sequence between the His tag and residues 49–523 of PlyB. All vectors were transformed into *E. coli* BL21 Codon Plus pLysS (DE3) (Novagen). PCR mutagenesis was used to substitute the single cysteine (residue 487) for an alanine (PlyB_C487A). The PlyB_C487A construct was used to generate the disulphide trap variants: TMH1-lock (C487A F138C H221C), TMH2 helix-lock (C487A Y166C G266C) and TMH2 strand-lock (C487A V277C K291C).

**PlyB Protein expression and refolding**

Transformed *E. coli* strains were cultured in 3 x 500 ml 2x YT media containing 100 mg ml^-1^ ampicillin and 34 mg ml^-1^ chloramphenicol at 37°C until an OD_600_ of 0.4 absorbance units had been reached. Protein expression was induced with the addition of IPTG to a final concentration of 1 mM, for 4 h at 310 K. The cells were then harvested by centrifugation (3,000*g*, 4°C, 20 min) and resuspended in 50 mM Tris-HCl pH 8.0 buffer containing 1% (w/v) Triton X-100, 1% (w/v) sodium deoxycholate, 100 mM NaCl, 5 mM MgCl_2_, 0.1 mg ml^-1^ DNaseI, 1 mg ml^-1^ lysozyme. The cellular suspension was incubated at room temperature for 30 min before the cells were disrupted by sonication. The lysed cell suspension was centrifuged (15,000g, 4°C, 30 min). The inclusion body pellet was resuspended in 50 mM Tris-HCl pH 8.0 buffer containing 0.5% (w/v) Triton X-100, 100 mM NaCl, 1 mM EDTA and centrifuged (15,000*g*, 4°C, 15 min). This first wash step was repeated until the inclusion body appeared white. A second wash step used 50 mM Tris-HCl pH 8.0 buffer containing 1 mM EDTA to wash the inclusion body using the above method.

After centrifugation, the inclusion body pellet was dissolved in 8 M Urea buffer containing 50 mM Tris-HCl pH 8.5, 10 mM β-mercaptoethanol and added dropwise over 16 hours into a 50 mM Tris-HCl pH 8.5 refold buffer containing 150 mM NaCl, 0.1 mM EDTA, 5% (v/v) glycerol.

**PlyB purification**

All untagged PlyB variants were purified using a refolding protocol adapted from Sakurai and colleagues [[2](#_ENREF_2)]. Refolded His tagged PlyB, was bound to a 5 mL IMAC column (Chelating Sepharose Fast Flow resin (Amersham Biosciences)), eluted in 50 mM Tris pH 8.5, 150 mM NaCl, 0.1 mM EDTA, 5% (v/v) glycerol, 300 mM imidazole and further purified using a Superdex 200 gel-filtration column (Amersham Biosciences) pre-equilibrated with 50 mM Tris pH 8.5, 150 mM NaCl, 0.1 mM EDTA, 5% (v/v) glycerol. TEV protease was used to remove the His tag and gel filtration used to further purify the untagged PlyB protein.

The disulphide lock mutants were monitored using non-reducing SDS-PAGE combined with haemolytic assays to ensure that 100% disulphide bond formation had occurred. In the case of the THM2 helix lock, samples were denatured by adding 6 M GuHCl and the sample split into two aliquots (sample A & B). One mM DTT was added to sample A to reduce all cysteines, while sample B retained all cysteines in their original state. Both samples were incubated at 65°C for 20 minutes, cooled to room temperature and treated with 2.5 mM chloroacetamide for 30 min in the dark. Excess chloroacetamide was quenched and disulfide bonds (sample B) were reduced and by addition of 8 mM DTT. 20 mM N-Ethylmaleimide (NEM) was used to alkylate all free and reduced cysteines (sample B). The two samples were digested with trypsin in solution, desalted and subjected to LC-MS analysis. The procedure was reversed whereby the first treatment was with NEM and the second with chloroacetamide [[4](#_ENREF_4)].

**Crystal structure determination for PlyA and PlyB**

Purified SeMet labeled PlyA was concentrated to ~10 mg ml^-1^ in 10 mM Tris-HCl at pH 8.4, 25 mM KCl, 10 mM β-mercaptoethanol. Diffraction-quality crystals of PlyA were obtained using the hanging-drop vapour-diffusion approach and 50 mM sodium citrate, pH 5.6, 12% (w/v) PEG3350, 0.2 M MgSO_4_ as the reservoir buffer. Crystals were cryo-cooled in the presence of 20% (v/v) MPD.

Purified TeV digested PlyB was concentrated to 4 mg ml^-1^ 50 mM Tris-HCl pH 8.0, 0.1 mM EDTA, 5% (w/v) glycerol, 50 mM NaCl. Diffraction-quality crystals of PlyB were obtained in hanging drop experiments using 0.2M NH_4_Ac, 0.1 M NaCitrate at pH 5.0 and 30% (w/v) PEG8000 as the reservoir buffer. Heavy atom derivatives were obtained by soaking PlyB crystals in solutions containing methyl mercury acetate, potassium iodide, or potassium tetracyanoplatinate(II) or platinum(II)-ammonium chloride heavy atoms.

Data were collected (0.95364 Å) on a single crystal of SeMet-PlyA. Multiple datasets were collected on native (0.9797 Å) and heavy atom soaked crystals (0.95665 Å) of PlyB. Data were merged and processed using MOSFLM [[5](#_ENREF_5)], POINTLESS [[6](#_ENREF_6)] and SCALA [[7](#_ENREF_7)]. Five per cent of the data sets were flagged as a validation set for calculation of the Rfree with neither a sigma nor a low-resolution cut-off applied to the data. Experimental phases were obtained by single-wavelength anomalous diffraction (SAD) for PlyA and multiple isomorphous replacement with anomalous scattering diffraction (MIRAS [[8](#_ENREF_8)]) for PlyB. These experiments used autoSHARP [[9](#_ENREF_9)]; heavy atom positions were located using SHELXC/SHELXD [[10](#_ENREF_10)] and refined using SHARP [[11](#_ENREF_11)]. The initial phases were improved by solvent flipping using SOLOMON [[12](#_ENREF_12)] and density modification using DM. An initial model of both proteins was generated using ARP/wARP [[13](#_ENREF_13)]. Model building was performed with COOT [[14](#_ENREF_14)] while refinement was performed with PHENIX [[15](#_ENREF_15)], REFMAC [[16](#_ENREF_16)] and autoBUSTER [[17](#_ENREF_17)]. Water molecules were added to each model when the Rfree reached 30%. Structural validation was performed using MolProbity [[18](#_ENREF_18)]. In the final PlyA and PlyB structures, 0.38% and 0.22% are in disallowed regions in the Ramachandran plot; the MolProbity scores are 1.18 and 1.11, which is in the 99th and 100th percentile of structures reported at the corresponding resolutions.

For the TMH1 lock (PlyB^C487A,F138C,H221C)^, protein was concentrated to 3.5 mg/mL and crystals obtained in 25% (w/v) PEG 8000, 0.1 M sodium cacodylate pH 5.5, 0.2 M ammonium sulfate using the hanging drop, vapour diffusion method. X-ray diffraction data were measured at the Australian Synchrotron (MX2 beamline) using an ADSC Quantum 210r detector image-plate detector. Data were collected to a resolution 2.2 Å for native TMH1 lock datasets. All data were indexed, integrated and scaled using XDS [[19](#_ENREF_19)]. Molecular replacement was used to determine the structure using the PHENIX program autoMR [[15](#_ENREF_15)]. The initial search model was PlyB_WT (PDB ID: 4OEJ). The model was refined using the PHENIX program PHENIX refine [[15](#_ENREF_15)].

**Titration based haemolytic assay**

Sheep red blood cells (SRBC) were resuspended to a concentration of 3 x 10^7^ cells/mL in 150 mM NaCl, 20 mM HEPES pH 7.2 (HBS). 200 uL of suspended cells were combined with solutions containing 0.1 mg/mL PlyA and variable concentrations of PlyB. PlyA and PlyB were pre-equilibrated on ice for 30 min before addition of the cell suspension. For reduced samples, DTT was added to PlyB dilutions at a final concentration of 10 mM for 30 min before combining with the cell suspension. Protein and blood were combined at 4 °C and then incubated at 24 °C for 30 min. The cells were then centrifuged at 12,000g for 10 s in a microcentrifuge. The supernatant was removed and the release of haemoglobin into the supernatant was measured (using absorbance at 405 nm) using a 96-well POLARstar OPTIMA Plate Reader (BMG Labtechnologies) in conjunction with the FLUOstar plate-reading program. Cell lysis was expressed as a percentage of maximum haemoglobin released by osmotic lysis in water.

**Red Blood Cell Pull-down Assay**

Stored SRBC were diluted 10-fold in HBS and washed for use in the pulldown assay. The assay consisted of a 50 μL reaction mix that contained either 20 μL of washed SRBC with a final concentration of 0.5 μg/mL PlyA. Negative controls contained the SRBC or PlyA in isolation. Each reaction mix was incubated for 30 min at 25°C, before pelleting the cells at 16,100g for 5 min. A sample of the supernatant (S) was put aside while the pellet was washed twice with a HBS solution. The final washed pellet (P) was resuspended in 20 mM HEPES to lyse the SRBC. For SDS-PAGE Western Blot analysis, the (P) sample was prepared by diluting the protein with 5x loading dye (0.025% (w/v) Bromophenol Blue, 5% (w/v) SDS, 5% (w/v) β-ME, 50% (w/v) glycerol) in a 1:4 ratio. An 8 μL of the (P) sample was run alongside 8 μL of the (S) sample on a 15% (w/v) SDS-PAGE. The gel was transferred to a nitrocellulose membrane via Western Transfer method, and was probed for PlyA using polyclonal rabbit anti-PlyA antibodies (WEHI Antibody Facility). After the membrane was developed, it was stripped and reprobed using anti-GAPDH monoclonal antibodies (Life Technologies^TM^, Australia), a protein present in SRBC.

**Real-time haemolytic assay – reactivation of trapped pleurotolysin prepores**

SRBC were resuspended to a concentration of 3 x 10^7^ cells/mL in 20 mM HEPES pH 7.2, 150 mM NaCl (HBS buffer) and were combined with PlyA (at a constant concentration 0.1 mg/mL) and the optimal concentration of the disulphide trapped PlyB mutant. A suspension of prelysed SRBC was measured as a reference for 100% lysis. For the TMH1 lock the optimal concentration was 73.4 pM, whereas for both of the TMH2 lock variants the optimal concentration was 13.1 nM. Protein and blood were combined and incubated at 25°C for 5 min. The cells were centrifuged at 16,100g for 5 min in a microcentrifuge. The supernatant was removed and the pellet was resuspended in HBS buffer. This wash step was repeated twice, to ensure any unbound proteins were removed and only assembled prepores were on the cell surface. The sample was aliquoted into 6 wells of a 96-well Falcon plate and inserted into a POLARstar OPTIMA Plate Reader (BMG Labtechnologies). Using the FLUOstar plate-reading program (BMG Labtechnologies) a program was written to read the absorbance caused by cell turbidity at 620 nm at set intervals for a set period of time. After the initial absorbance reading, DTT was dispensed into three of the wells (final concentration was 10 mM). Cell lysis was measured by the decrease in turbidity.

**EM sample preparation and data acquisition**

Sphingomyelin/cholesterol liposomes (1:1 molar ratio, final concentration 2–4 mM in 50 mM NaCl, 20 mM HEPES pH 7.4) were prepared using an Avanti^®^ Mini-Extruder with 80 nm polycarbonate membranes according to the manufacturer’s instructions (Avanti Polar Lipids).

Ply WT pores and engineered disulphide bond oligomers trapped on liposomes were prepared as described in Tomita et al. [[20](#_ENREF_20)] with the following modifications: PlyA was first added to sphingomyelin/cholesterol liposomes at a molar ratio of 1:2000–5000 protein to lipid in the above buffer. After 1–5 min incubation at room temperature, WT or mutant PlyB was added to the mixture at a molar ratio of 1:1–2 PlyB to PlyA. The mixture was incubated at 40°C or room temperature for 10–40 min after which 3.5 μL were placed on negatively glow discharged lacey grids (Elektron Technology/Agar Scientific) and vitrified in liquid ethane using a Vitrobot (FEI). Blotting was carried out at 36°C and 80% humidity. For negative stain EM, mixtures were diluted ~5 times with the above buffer and 3 μL were applied to glow-discharged carbon-coated copper grids (Elektron Technology/Agar Scientific) and stained with 1% (w/v) uranyl acetate.

Low dose images of negatively stained pleurotolysin pores and prepores on liposomes were recorded with a Gatan 4k×4k CCD camera (15 μm/pixel) on a Tecnai F20 microscope (FEI) operating at an accelerating voltage of 200 keV. These were used to analyse the symmetry of pleurotolysin oligomers and for initial quality screening of the preparation used for cryo-EM reconstruction.

Low dose cryo-EM images of pleurotolysin pores and prepores on liposomes were recorded with a Gatan 4k×4k CCD camera (15 μm/pixel) on a Tecnai Polara microscope (FEI) operating at an accelerating voltage of 300 keV and a magnification of 76148x (2.0 Å/pixel) at the specimen level (defocus range 1.3–3.5 μm).

**Image processing**

Single-particle image analysis was performed using IMAGIC [[21](#_ENREF_21)] and SPIDER [[22](#_ENREF_22)] software packages. Micrograph defocus parameters were determined with the MRC program CTFFIND [[23](#_ENREF_23)] and phases were corrected for effects of the contrast transfer function. EMAN/Boxer [[24](#_ENREF_24)] was used for selection of individual images.

**Symmetry analysis of negatively stained pleurotolysin oligomers**

Selected individual end views (n=730) of complete oligomeric rings of negatively stained WT pleurotolysin were translationally aligned to their rotationally averaged sum. Multivariate statistical analysis (MSA) indicated 13-fold symmetry for more than 75% of end views, 12-fold for ~15%; 11- and 14-fold symmetries were also detected for less than 5% of examined images each (data not shown). Similar proportions of 13-fold and 12-fold symmetries were estimated when oligomers of disulphide bond-trapped prepore states were analysed using the same approach; 11- and 14-fold symmetries were not detected, most likely because much smaller data sets were selected (~300 each).

**Cryo-EM structure determination of pleurotolysin pore and disulphide lock prepores**

Pore end views (n = 2,440) were aligned and symmetry estimated as described above for negatively stained rings. A collection of 14,700 individual images of pore side views were aligned and classified according to their diameter and tilt by MSA. Particle orientations were refined in multiple cycles of multi-reference alignment (MRA) and MSA. Class averages with the lowest variance were used to obtain low-resolution density maps by angular reconstitution [[21](#_ENREF_21)] using 12- and 13-fold symmetries and the resulting 3D reconstructions were used as initial models for simultaneous projection matching in SPIDER [[22](#_ENREF_22)]. This allowed selection of 11,000 pleurotolysin pore side views with estimated 13-fold symmetry. These images were split randomly into two data sets, which were analysed again independently: views were aligned and classified according to their tilt by MSA, particle orientations were refined in multiple cycles of MRA and MSA and class averages with lowest variance were used to obtain low-resolution density maps with 13-fold symmetry by angular reconstitution for each data set. These 3D reconstructions were used as initial models for iterative competitive projection matching in SPIDER with resolution cut off estimated after each iteration using gold standard Fourier shell correlation (FSC) plots [[25](#_ENREF_25),[26](#_ENREF_26)]. The final reconstruction with 13-fold symmetry comprised 8,770 (~80%) particles from both datasets resulting in structures at 10.5 Å resolution estimated by gold standard FSC with the 0.143 criterion (Figure S9A). The map was filtered between 30–11 Å to correct for the over-representation of low-frequency information and to remove high-frequency noise. The low-frequency components were reduced to 15% of their original amplitudes.

A similar approach using side views only was applied to determine structures of disulphide bond trapped states. However, ~5 times smaller datasets (1,700-2,700 particles) were collected due to increased aggregation of protein and liposomes in these mutant preparations. Because of this limitation we could not split each dataset and analyse the two halves independently; conventional FSCs [[26](#_ENREF_26)] were used instead to estimate resolution. The final reconstructions with 13-fold symmetry (Figure 5) comprised 1,150 particles for TMH1 lock, 722 particles for TMH2 helix lock and 1,110 particles for TMH2 strand lock prepores, resulting in corresponding structures at 15 Å, 17 Å, and 14 Å resolution estimated by FSC with the 0.5 correlation criterion (Figure S9, B to D).

**Atomic structure fitting and refinement**

Initial fitting of PlyA and PlyB atomic coordinates into the cryo-EM maps was done manually using Chimera [[27](#_ENREF_27)]. In the pore map, the position of PlyB is clearly recognizable in the upper part of each subunit, while the V-shaped density at the base of each asymmetric unit accommodates two PlyA molecules. The hand of the pore map was chosen because it provided a better fit for PlyB C-terminal trefoil (cross-correlation 0.40 vs 0.27).

The positions of PlyB subdomains were refined without TMH1 and TMH2, because these transmembrane regions are refolded to form the β-barrel of the pore. The missing residues (49-57) in the N-terminus of native PlyB structure were modelled using MODELLER [[28](#_ENREF_28)] based on the N-terminal of PlyB^C487A,F138C,H221C^ (TMH1 lock) X-ray structure.

The best fits were further refined with Flex-EM [[29](#_ENREF_29)] using a multistep procedure via simulated annealing rigid-body dynamics. In the first step each map was box-segmented around three adjacent asymmetric units containing six PlyA and three PlyB molecules and the EM density corresponding to the β-barrel was erased (using Chimera). Only loops between residues 397 and 407, 424 and 430, and 477 and 489 on the interface of the PlyB molecules and N-terminus (residues 49-71) were treated flexibly, to relieve any clashes between neighbouring subunits. Within PlyB multiple combinations of rigid bodies [[30](#_ENREF_30)] were refined and converged to the same fit. Once the central asymmetric unit was refined, the top of the pore was rebuilt using C13 symmetry in Chimera. Using MODELLER, the resulting model was then combined with the β-barrel model with the strands tilted 20º to the pore axis [[31](#_ENREF_31)] (Figure S2). Next, the map was re-segmented around three asymmetric units including the β-barrel. Loop refinement was then performed on the loops connecting the top of the MACPF domain (residues 152-156, 216-220, 275-279, 341-346) and the corresponding strands from the modelled barrel (only on the central asymmetric unit). One thousand loops were generated and the top 20 models were chosen based on the cross-correlation coefficient with the map. These loops were re-ranked based on the Discrete Optimized Protein Energy (DOPE) statistical potential score [[28](#_ENREF_28)]. Due to steric clashes with the barrel, further refinement using Flex-EM was performed on the helix-turn-helix (HTH) motif (residues 298-313). After refinement of the central asymmetric unit, the pore was rebuilt with C13 symmetry in Chimera to give the final pore model.

**Analysis** **of pore opening**

To analyse the rigid- body shift and rotation of the MACPF β-sheet in the pore model (residues 152-162, 205-219, 278-290, 335-345, red in Figure 1B) with respect to the monomer crystal structure, we used the domain-orientation score [[29](#_ENREF_29)]. Firstly, the structures were aligned by superimposing the top of the MACPF domain of the pore model with the X-ray structure (blue, Figure 3C) using least-squares superposition as implemented in MODELLER. Then the domain-orientation score was calculated from the two positions of the MACPF β-sheet region, yielding a translation of ~13 Å (of the centre of mass) and rotation angle of ~70°.

To analyse the conformation of PlyB in the prepore maps, which were determined at lower resolution (14-17 Å), we used the monomer structure and pore model to define the end points (Figure S8A). Based on the domain-orientation score, two series of angular sweeps (starting from each of the end points) of the MACPF β-sheet were generated in steps of 0.5 Å translation and 1° rotation around the centre of mass (Figure S8B). Using MODELLER, each sampled conformation of the MACPF β-sheet was combined with the PlyB monomer structure or with the pore model (excluding the TMH1, TMH2 and C-terminal trefoil regions and their connecting linkers), resulting in a total of ~4,600 models (Figure S8C). Each prepore map was segmented around three adjacent asymmetric units and the models were rigidly fitted into the central asymmetric units (Figure S8D) based on the respective reference structure (the monomer structure and the pore model). For each model the goodness-of-fit of the MACPF β-sheet was assessed based on the segment**-**based cross-correlation score [[32](#_ENREF_32)] (SCCC). The top 20 models were re-ranked based on the MACPF β-sheet region local DOPE score [[28](#_ENREF_28)] (Figure 6, S8E, Table S4).

**Liposome preparation**

Liposomes used for fluorescence experiments were composed of porcine brain sphingomyelin (45 mol %) and cholesterol (55 mol %) (Avanti Polar Lipids) and generated via extrusion using an Avestin extruder (Ottawa, ON) as previously described [[33](#_ENREF_33)]. Liposomes used for quenching studies were made similarly except that 5 mol % of the sphingomyelin was replaced by nitroxide-labelled phospholipid [1-palmitoyl-2-stearoyl-(12-doxyl)*-sn-*glycero-3-phosphocholine] (Avanti Polar Lipids).

**Modification of cysteine-substituted PlyB with IANBD**

To test the hypothesis that the α-helical bundles of pleurotolysin unravel to form extended transmembrane β-hairpins, we carried out fluorescence spectroscopy studies similar to those used to identify the CDC TMHs [[33](#_ENREF_33),[34](#_ENREF_34)]. Single cysteine substitutions were generated along the primary structure of TMH1, the mutant proteins purified and the cysteine sulfhydryl modified with the sulfhydryl-specific, environmentally sensitive fluorescent probe NBD [[33](#_ENREF_33),[34](#_ENREF_34)]. The emission of each NBD-modified mutant was determined in the absence and presence of liposomal membranes and exhibited an alternating pattern of emission (Figure S1) consistent with a membrane-spanning amphipathic β-hairpin structure [[33](#_ENREF_33),[34](#_ENREF_34)]. The membrane location of the NBD probes was confirmed by placing a collisional quencher within the bilayer core: the NBD emission is quenched if the amino acid sidechain positioned NBD within the membrane whereas it would not be quenched if it faced the fluid filled pore (Figure S1).

**Fluorescence Measurements**

All fluorescence intensity measurements were performed using a Fluorolog fluorometer (JY Horiba Inc.) as previously described [[33](#_ENREF_33)]. A typical NBD emission experiment contained PlyB (85 pmol) and PlyA (294 pmol) in 2.5 ml HBS. Emission spectra were taken before and after addition of 20 μl of liposomes.

1. Van Duyne GD, Standaert RF, Karplus PA, Schreiber SL, Clardy J (1993) Atomic structures of the human immunophilin FKBP-12 complexes with FK506 and rapamycin. J Mol Biol 229: 105-124.

2. Sakurai N, Kaneko J, Kamio Y, Tomita T (2004) Cloning, expression, and pore-forming properties of mature and precursor forms of pleurotolysin, a sphingomyelin-specific two-component cytolysin from the edible mushroom Pleurotus ostreatus. Biochim Biophys Acta 1679: 65-73.

3. Law RH, Irving JA, Buckle AM, Ruzyla K, Buzza M, et al. (2005) The high resolution crystal structure of the human tumor suppressor maspin reveals a novel conformational switch in the G-helix. J Biol Chem 280: 22356-22364.

4. Held JM, Danielson SR, Behring JB, Atsriku C, Britton DJ, et al. (2010) Targeted quantitation of site-specific cysteine oxidation in endogenous proteins using a differential alkylation and multiple reaction monitoring mass spectrometry approach. Mol Cell Proteomics 9: 1400-1410.

5. Leslie AGW (1992) CCP4+ESF-EACMB. Newslett Protein Crystallogr. pp. 27-33.

6. Evans P (2006) Scaling and assessment of data quality. Acta Crystallogr D Biol Crystallogr 62: 72-82.

7. Evans PR (1993) Data reduction. Proceedings of CCP4 Study Weekend, on Data Collections & Processing: 114-122.

8. Dauter Z (2002) New approaches to high-throughput phasing. Curr Opin Struct Biol 12: 674-678.

9. Vonrhein C, Blanc E, Roversi P, Bricogne G (2007) Automated structure solution with autoSHARP. Methods Mol Biol 364: 215-230.

10. Sheldrick GM (2010) Experimental phasing with SHELXC/D/E: combining chain tracing with density modification. Acta Crystallogr D Biol Crystallogr 66: 479-485.

11. de La Fortelle E, Bricogne G (1997) [27] Maximum-likelihood heavy-atom parameter refinement for multiple isomorphous replacement and multiwavelength anomalous diffraction methods. In: Charles W. Carter, Jr., editor. Methods in Enzymology: Academic Press. pp. 472-494.

12. Abrahams JP, Leslie AG (1996) Methods used in the structure determination of bovine mitochondrial F1 ATPase. Acta Crystallogr D Biol Crystallogr 52: 30-42.

13. Langer G, Cohen SX, Lamzin VS, Perrakis A (2008) Automated macromolecular model building for X-ray crystallography using ARP/wARP version 7. Nat Protoc 3: 1171-1179.

14. Emsley P, Cowtan K (2004) Coot: model-building tools for molecular graphics. Acta Crystallogr D Biol Crystallogr 60: 2126-2132.

15. Adams PD, Afonine PV, Bunkoczi G, Chen VB, Davis IW, et al. (2010) PHENIX: a comprehensive Python-based system for macromolecular structure solution. Acta Crystallogr D Biol Crystallogr 66: 213-221.

16. Murshudov GN, Vagin AA, Dodson EJ (1997) Refinement of macromolecular structures by the maximum-likelihood method. Acta Crystallogr D Biol Crystallogr 53: 240-255.

17. Bricogne G. BE, Brandl M., Flensburg C., Keller P., Paciorek W.,, Roversi P SA, Smart O.S., Vonrhein C., Womack T.O. (2011) BUSTER version X.Y.Z. Cambridge, United Kingdom: Global Phasing Ltd.

18. Chen VB, Arendall WB, 3rd, Headd JJ, Keedy DA, Immormino RM, et al. (2010) MolProbity: all-atom structure validation for macromolecular crystallography. Acta Crystallogr D Biol Crystallogr 66: 12-21.

19. Kabsch W (2010) Xds. Acta Crystallogr D Biol Crystallogr 66: 125-132.

20. Tomita T, Noguchi K, Mimuro H, Ukaji F, Ito K, et al. (2004) Pleurotolysin, a novel sphingomyelin-specific two-component cytolysin from the edible mushroom Pleurotus ostreatus, assembles into a transmembrane pore complex. J Biol Chem 279: 26975-26982.

21. van Heel M, Harauz G, Orlova EV, Schmidt R, Schatz M (1996) A new generation of the IMAGIC image processing system. J Struct Biol 116: 17-24.

22. Frank J, Radermacher M, Penczek P, Zhu J, Li Y, et al. (1996) SPIDER and WEB: processing and visualization of images in 3D electron microscopy and related fields. J Struct Biol 116: 190-199.

23. Crowther RA, Henderson R, Smith JM (1996) MRC image processing programs. J Struct Biol 116: 9-16.

24. Ludtke SJ, Baldwin PR, Chiu W (1999) EMAN: semiautomated software for high-resolution single-particle reconstructions. J Struct Biol 128: 82-97.

25. Henderson R, Sali A, Baker ML, Carragher B, Devkota B, et al. (2012) Outcome of the first electron microscopy validation task force meeting. Structure 20: 205-214.

26. Scheres SH, Chen S (2012) Prevention of overfitting in cryo-EM structure determination. Nat Methods 9: 853-854.

27. Pettersen EF, Goddard TD, Huang CC, Couch GS, Greenblatt DM, et al. (2004) UCSF Chimera--a visualization system for exploratory research and analysis. J Comput Chem 25: 1605-1612.

28. Shen MY, Sali A (2006) Statistical potential for assessment and prediction of protein structures. Protein Sci 15: 2507-2524.

29. Topf M, Lasker K, Webb B, Wolfson H, Chiu W, et al. (2008) Protein structure fitting and refinement guided by cryo-EM density. Structure 16: 295-307.

30. Pandurangan AP, Topf M (2012) Finding rigid bodies in protein structures: Application to flexible fitting into cryoEM maps. J Struct Biol 177: 520-531.

31. Reboul CF, Mahmood K, Whisstock JC, Dunstone MA (2012) Predicting giant transmembrane beta-barrel architecture. Bioinformatics 28: 1299-1302.

32. Pandurangan AP, Shakeel S, Butcher SJ, Topf M (2014) Combined approaches to flexible fitting and assessment in virus capsids undergoing conformational change. J Struct Biol 185: 427-439.

33. Shepard LA, Heuck AP, Hamman BD, Rossjohn J, Parker MW, et al. (1998) Identification of a membrane-spanning domain of the thiol-activated pore-forming toxin Clostridium perfringens perfringolysin O: an alpha-helical to beta-sheet transition identified by fluorescence spectroscopy. Biochemistry 37: 14563-14574.

34. Shatursky O, Heuck AP, Shepard LA, Rossjohn J, Parker MW, et al. (1999) The mechanism of membrane insertion for a cholesterol-dependent cytolysin: a novel paradigm for pore-forming toxins. Cell 99: 293-299.
