# Supplementary material for: Conformational Changes during Pore Formation by the Perforin-Related Protein Pleurotolysin
Source: PLoS Biol. 2015 Feb 5;13(2):e1002049. doi: 10.1371/journal.pbio.1002049 (PMC4318580; doi:10.1371/journal.pbio.1002049)
Supplement: S1 Table — (DOCX) [file pbio.1002049.s012.docx]

**Table S1** PlyA data collection, phasing and refinement statistics (PDB ID 4OEB).

|  | Seleno Methionine |
| --- | --- |
| **Data Collection** |  |
| Space group | P62 |
| Cell dimensions  *a, b, c* (Å) | 132.0, 132.0, 65.4 |
| α, β, γ (˚) | 90, 90, 120 |
| Resolution range (Å) | 29.45 (2.00)* |
| *R*_merge_ | 0.113 (0.561) |
| *R*_pim_ | 0.017 (0.085) |
| *I*/σ*I* | 49.9 (11.5) |
| Completeness (%) | 100 (100) |
| Redundancy | 88.1 (87.5) |
|  |  |
| **Refinement** |  |
| Resolution (Å) | 43.11 (1.85) |
| No. reflections (work/free) | 3695/205 |
| *R*_work_/*R*_free_ | 0.1724/0.1982 |
| No Atoms  Protein | 4166 |
| Water | 498 |
| Ligand/ion | 20 |
|  |  |
| B-factors  Protein | 34.85 |
| Ligand/ion | 38.35 |
| Water | 42.53 |
| R.m.s. deviations  Bond lengths (Å) | 0.010 |
| Bond Angles (^o^) | 1.15 |

*Highest resolution shell is shown in parenthesis.
