# Supplementary material for: Conformational Changes during Pore Formation by the Perforin-Related Protein Pleurotolysin
Source: PLoS Biol. 2015 Feb 5;13(2):e1002049. doi: 10.1371/journal.pbio.1002049 (PMC4318580; doi:10.1371/journal.pbio.1002049)
Supplement: S2 Table — (DOCX) [file pbio.1002049.s013.docx]

**Table S2** PlyB data collection, phasing and refinement statistics (PDB ID 4OEJ).

|  | Native 1 | (NH_4_)_2_PtCl_4_ | K_2_Pt(CN)_4_ | (C_2_H_5_HgO)HPO_2_ | KI | Native 2 |
| --- | --- | --- | --- | --- | --- | --- |
| **Data collection** | | | | | | |
| Space group | P 3_1_ 2 1 | P 3_1_ 2 1 | P 3_1_ 2 1 | P 3_1_ 2 1 | P 3_1_ 2 1 | P 3_1_ 2 1 |
| Cell dimensions  *a, b, c* (Å) | 71.2, 71.2, 175.8 | 71.0, 71.0, 175.6 | 71.2, 71.2, 175.6 | 71.2, 71.2, 174.7 | 71.6, 71.6, 179.4 | 71.6, 71.6, 174.9 |
| α, β, γ (˚) | 90, 90, 120 | 90, 90, 120 | 90, 90, 120 | 90, 90, 120 | 90, 90, 120 | 90, 90, 120 |
| Resolution range (Å) | 35.8 (3.0)* | 87.7 (2.8) | 33 (2.8) | 35.6 (3.4) | 35.8 (3.0) | 87.4 (2.2) |
| *R*_merge_ | 9.2 (40.2) | 12.1 (76.7) | 11.6 (70.6) | 10.2 (15.7) | 22.1 (61.3) | 7.7 (66.9) |
| *R*_pim_ | 1.8 (7.7) | 4.1 (33.6) | 3.8 (21.6) | 3.4 (5.0) | 7.2 (19.7) | 2.6 (23.3) |
| *I*/σ*I* | 39.2 (9.4) | 14.6 (2.2) | 17.3 (3.8) | 21.6 (13.8) | 11.9 (3.8) | 18.8 (3.9) |
| Completeness (%) | 100 (100) | 99.9 (99.4) | 99.9 (100) | 99.9 (100) | 100 (100) | 100 (100) |
| Redundancy | 28.5 (27.4) | 10 (6.9) | 11.8 (12.1) | 11.6 (12.0) | 28.5 (27.4) | 10.2 (9.9) |
| **Refinement** | | | | | | |
| Resolution (Å) | 26.56 (2.20) | | | | | |
| No. reflections (work/free) | 2807/117 | | | | | |
| *R*_work_/*R*_free_ | 0.1892/0.2213 | | | | | |
| No. Atoms |  | | | | | |
| Protein | 3475 | | | | | |
| Ligand/ion | 41 | | | | | |
| Water | 160 | | | | | |
| B-factors |  | | | | | |
| Protein | 59.60 | | | | | |
| Ligand/ion | 76.12 | | | | | |
| Water | 54.06 | | | | | |
| R.m.s. deviations |  | | | | | |
| Bond lengths (Å) | 0.008 | | | | | |
| Bond Angles (^o^) | 1.07 | | | | | |

*Highest resolution shell is shown in parenthesis.
