# Supplementary material for: Conformational Changes during Pore Formation by the Perforin-Related Protein Pleurotolysin
Source: PLoS Biol. 2015 Feb 5;13(2):e1002049. doi: 10.1371/journal.pbio.1002049 (PMC4318580; doi:10.1371/journal.pbio.1002049)
Supplement: S3 Table — (DOCX) [file pbio.1002049.s014.docx]

**Table S3** PlyB TMH1-lock data collection and refinement statistics (MR) (PDB ID 4OV8).

|  | Native |
| --- | --- |
| **Data collection** | |
| Space group | P 3_1_ 2 1 |
| Cell dimensions  *a, b, c* (Å) | 71.18, 71.18, 174.68 |
| α, β, γ (˚) | 90, 90, 120 |
| Resolution range (Å) | 174.68 (2.15)* |
| *R*_merge_ | 0.091 (0.861) |
| *R*_pim_ | 0.031 (0.307) |
| *I/*σ*I* | 16.5 (3.0) |
| Completeness (%) | 100 (100) |
| Redundancy | 9.5 (8.4) |
|  | |
| **Refinement** | |
| Resolution (Å) | 61.644 (2.15) |
| No. reflections (work/free) | 28729/1456 |
| *R*_work_/*R*_free_ | 0.2072/0.2426 |
| No. Atoms |  |
| Protein | 3530 |
| Ligand/ion | 22 |
| Water | 174 |
| B-factors |  |
| Protein | 59.302 |
| Ligand/ion | 89.563 |
| Water | 48.03 |
| r.m.s deviations |  |
| Bond lengths (Å) | 0.004 |
| Bond Angles (^o^) | 0.821 |

*Highest resolution shell is shown in parenthesis.
