# Supplementary material for: Conformational Changes during Pore Formation by the Perforin-Related Protein Pleurotolysin
Source: PLoS Biol. 2015 Feb 5;13(2):e1002049. doi: 10.1371/journal.pbio.1002049 (PMC4318580; doi:10.1371/journal.pbio.1002049)
Supplement: S4 Table — (DOCX) [file pbio.1002049.s015.docx]

**Table S4. Domain-orientation scores [30] (DOS, translation and rotation parameters) of the 20 best fitting sheet conformations for TMH1 lock, TMH2 helix lock, and TMH2 strand lock prepores (Figure 6).**

| Index* | TMH1 lock prepore | | TMH2 helix lock prepore | | TMH2 strand lock prepore | |
| --- | --- | --- | --- | --- | --- | --- |
|  | Distance, Å | Angle, degree | Distance, Å | Angle, degree | Distance, Å | Angle, degree |
| 1 | 9.3 | 46.6 | 5.7 | 32.5 | 9.3 | 46.6 |
| 2 | 10.1 | 54.1 | 4.5 | 29.0 | 9.4 | 48.9 |
| 3 | 9.9 | 52.1 | 3.7 | 29.4 | 9.3 | 43.7 |
| 4 | 9.3 | 43.7 | 6.5 | 35.0 | 9.6 | 46.2 |
| 5 | 9.6 | 46.2 | 5.4 | 28.9 | 9.3 | 40.2 |
| 6 | 9.9 | 80.8 | 4.3 | 29.2 | 8.4 | 39.3 |
| 7 | 9.3 | 40.2 | 6.1 | 34.9 | 8.5 | 71.5 |
| 8 | 9.3 | 59.7 | 5.7 | 36.6 | 8.8 | 43.6 |
| 9 | 10.2 | 52.7 | 4.0 | 24.2 | 9.3 | 59.7 |
| 10 | 10.4 | 54.5 | 5.8 | 49.9 | 9.1 | 53.6 |
| 11 | 9.4 | 42.5 | 6.8 | 36.4 | 8.9 | 47.3 |
| 12 | 9.7 | 48.4 | 4.0 | 55.6 | 9.4 | 42.5 |
| 13 | 9.8 | 60.5 | 5.8 | 34.2 | 9.2 | 52.1 |
| 14 | 9.3 | 45.7 | 3.5 | 23.3 | 8.9 | 42.6 |
| 15 | 9.5 | 53.8 | 4.9 | 52.3 | 9.3 | 45.7 |
| 16 | 9.7 | 50.5 | 6.5 | 67.3 | 9.0 | 47.4 |
| 17 | 9.2 | 49.0 | 5.3 | 32.0 | 9.3 | 51.5 |
| 18 | 10.3 | 52.8 | 6.0 | 35.0 | 9.0 | 47.6 |
| 19 | 9.3 | 51.5 | 7.6 | 42.1 | 8.3 | 64.8 |
| 20 | 9.9 | 65.6 | 6.4 | 35.7 | 9.2 | 52.0 |
| Mean ± standard deviation | 9.67 ± 0.37 | 52.5 ± 9.1 | 5.4 ± 1.1 | 37.1 ± 11.1 | 9.0 ± 0.35 | 49.3 ± 8.1 |

*Models were selected based on the segment**-**based cross-correlation score (SCCC [34]) and re-ranked based on statistical potentials of mean force score (DOPE [43])_._
